# Children's First Experience of Taking Anabolic-Androgenic Steroids can Occur before Their 10th Birthday: A Systematic Review Identifying 9 Factors That Predicted Doping among Young People

**DOI:** 10.3389/fpsyg.2017.01015

**Published:** 2017-06-20

**Authors:** Adam R. Nicholls, Ed Cope, Richard Bailey, Katrin Koenen, Detlef Dumon, Nikolaos C. Theodorou, Benoit Chanal, Delphine Saint Laurent, David Müller, Mar P. Andrés, Annemarie H. Kristensen, Mark A. Thompson, Wolfgang Baumann, Jean-Francois Laurent

**Affiliations:** ^1^School of Life Sciences, University of HullHull, United Kingdom; ^2^International Council of Sport Science and Physical EducationBerlin, Germany; ^3^KEA Fair Play Code HallasAthens, Greece; ^4^Agence Française de Lutte Contre le DopageParis, France; ^5^Nationale Anti-Doping Agentur Austria GmbHWien, Austria; ^6^Agencia Española de Protección de la Salud en el DeporteMadrid, Spain; ^7^Anti Doping DenmarkBroendby, Denmark; ^8^The Association for International Sport for AllFrankfurt, Germany

**Keywords:** performance enhancing drugs, gender differences, age differences, nutritional supplements, entourage, ethnicity, adolescents, attitudes

## Abstract

Taking performance-enhancing drugs (PEDs) can cause serious and irreversible health consequences, which can ultimately lead to premature death. Some young people may take PEDs without fully understanding the ramifications of their actions or based on the advice from others. The purpose of this systematic review was to identify the main factors that predicted doping among young people. The literature was systematically reviewed using search engines, manually searching specialist journals, and pearl growing. Fifty-two studies, which included 187,288 young people aged between 10 and 21 years of age, 883 parents of adolescent athletes, and 11 adult coaches, who were interviewed regarding young athletes, were included in this review. Nine factors predicted doping among young people: gender; age; sports participation; sport type; psychological variables; entourage; ethnicity; nutritional supplements; and health harming behaviors. In regards to psychological variables, 22 different constructs were associated with doping among young people. Some psychological constructs were negatively associated with doping (e.g., self-esteem, resisting social pressure, and perfectionist strivings), whereas other were positively associated with doping (e.g., suicide risk, anticipated regret, and aggression). Policy makers and National Anti-Doping Organizations could use these findings to help identify athletes who are more at risk of doping and then expose these individuals to anti-doping education. Based on the current findings, it also appears that education programs should commence at the onset of adolescence or even late childhood, due to the young age in which some individuals start doping.

## Introduction

According to the World Anti-Doping Agency's (WADA) most recent guide, doping is defined by the occurrence of at least one or more anti-doping rule violation (ADRV). There are 10 ADRVs, which included: (1) the presence of prohibited substances (e.g., Anabolic Androgenic Steroids; AAS), or its metabolites or markers within an athlete's sample; (2) use or attempted use of a banned substance or method (e.g., intravenous infusions at a rate of more than 150 ml per 6 h), (3) evading, failing, or refusing to provide a sample, (4) missing three tests within 12 months, (5) tampering or attempting to tamper with samples, (6) possessing a banned substance or method, (7) trafficking or attempt to traffic banned substances or methods, (8) administering banned substances or attempting to administer banned substances to athletes, (9) assisting or encouraging others to take banned substances, and (10) associating with individuals who are currently banned. ADRVs regularly feature in the media due to high profile cases with famous individual athletes, teams, or national organizations. Most of the cases portrayed in the media involve elite adult athletes, but it would be incorrect to assume that doping occurs exclusively among this population. The European School Survey Project on Alcohol and Other Drugs report (ESPAD, 2015) surveyed 96,043 young people from 35 European countries, and their findings revealed that around 1% of school pupils took AAS. The prevalence of doping varied from country to country, and was as high as 4% in Bulgaria. Furthermore, in Bulgaria 7% of young males abused AAS and 5% of Cypriot young males used AAS. The previous ESPAD report (ESPAD, 2011) revealed that doping violations occurred among young athletes participating in grassroots sports, too. It is therefore reasonable to suppose that a minority of young people take PEDs, regardless of their level of sport. Doping is a cause for concern because it represents a threat to sporting values, and poses a risk to players' health and well-being (Commission of the European Communities, 2007). Sport is formally framed by values, such as fair play, fair competition, respect for rule, and integrity. Doping is typically counted as cheating precisely because it threatens what is intrinsically valuable about sport, or what the WADA Code calls “the spirit of sport” (WADA, 2015). Doping also poses a serious threat to the lives of individuals who abuse PEDs (Nicholls et al., 2017b). PEDs can cause physical health problems such as liver, heart, and kidney damage (Bird et al., 2016), and are associated with a 2-to-4-fold increased risk of suicide (Lindqvist et al., 2013). These serious side effects could be a result of the supraphysiological consumption rates of PEDs, which are often above and beyond the levels for which these drugs were intended. Many of the physical side effects are irreversible, and can ultimately cause premature death (Bird et al., 2016). High quality studies on doping among young people now appear frequently in academic journals, but reviews regarding doping among young people are scarce. The review by Backhouse et al. (2007) identified eight studies among young people, concluding that most adolescent athletes possessed a negative attitude toward PEDs. Further, most young people believed that doping was dangerous to their health. More contemporary studies examined the relationship between doping and different psychological constructs (e.g., anticipated regret, aggression, and perfectionism), and revealed a variety of different psychological constructs that predicted doping, which were not included in the Backhouse et al. review. Researchers have used a variety of different measures, which can make comparing findings from studies difficult. For example, Bloodworth et al. (2012) used a “modified version of a questionnaire used by UK Sport in its 2005 Drug-Free Sport survey” (p. 295). However, the authors omitted to report the modifications made, the underpinning theoretical framework, or the reliability of scale. Another study invited athletes to respond to a stem proposition in which they gave their views of PEDs (e.g., bad/good, useless/useful, harmful/beneficial, or unethical/ethical; Barkoukis et al., 2015). These are two examples of researchers using different approaches to assess either doping prevalence or factors that influence doping. A systematic review, which takes account of different methods used to assess factors that predict doping among young people and provides an update on Backhouse et al.'s (2007) report is, therefore, warranted. For the purpose of this review, young people are classified as either children (aged 4 to 11) or adolescents (aged 12 and 21, following Weiss and Bredemeier, 1983). Targeting young people is especially important because this is the time when values and attitudes typically develop, and then take shape (Döring et al., 2015; Cieciuch et al., 2016; Kjellström et al., 2017). Attitudes appear particularly important in relation to doping behavior; a recent meta-analysis by Ntoumanis et al. (2014) showed that attitudes predicted the use of PEDs. It should be noted, however, that Ntoumanis' meta-analysis included both adolescents and adults. Providing an accurate representation of factors that predict doping among young people could help policy makers, governing bodies for sport, and National Anti-Doping Organizations (NADOs) identify the young people most at risk of taking PEDs, and offer appropriate support. Consequently, the purpose of this paper is to identify the factors that predicted doping among young people aged 21 years and younger.

## Methods

### Information sources and search strategy

In accordance with Nicholls et al. (2016), the authors utilized three distinct search strategies to identify appropriate studies: accessing search engines, manually searching specialist peer reviewed journals, and ‘Pearl Growing’ (Hartley, 1990). Medline, PsycINFO, PubMed and SportDISCUS electronic databases, as well as Google Scholar, and the research networking website, Research Gate, were all searched for appropriate studies, with no date limits. A preparatory meeting of all authors, on the 20th of February 2017, generated the list of keywords (i.e., “anabolic,” “androgenic steroids,” “blood doping,” “blood transfusion,” “doping,” “drugs,” “gene doping,” “growth hormone,” “performance enhancing drugs,” “nutritional supplements,” “pharmaceuticals,” “stimulants,” and “substance”) were identified and then used in this search. These words were used in conjunction with “adolescents”. “athletes,” “children,” “grassroots sports,” “juniors,” “mass participation,” “participation,” “physical activity,” “recreational,” “sport,” “sports players,” “sport for all,” “young people,” and “youth”. The first, second, and 12th authors independently searched the following specialist journals, which had a history of publishing articles on PED usage: *Addiction* (1903 to 2017), *Archives of Pediatrics and Adolescent Medicine* (2000 to 2017), *British Journal of Sports Medicine* (1964 to 2017), *Clinical Journal of Sports Medicine* (1991 to 2017), *European Journal of Clinical Pharmacology* (1968 to 2017), *International Journal of Sport and Exercise Psychology* (2003 to 2017), *International Journal of Sport Psychology* (1994 to 2017), *International Journal of Sport of Sports Medicine* (1980 to 2017), *Journal of Adolescent Health* (1980 to 2017), *Journal of Applied Sport Psychology* (1989 to 2017), *Journal of Child and Adolescent Substance Abuse* (1990 to 2017), *Journal of Clinical Sport Psychology* (2007 to 2017), *Journal of Drug Education* (1971 to 2017), *Journal of Drug Issues* (1971 to 2017), *Journal of Health Psychology*, (1996 to 2017), *Journal of Science and Medicine in Sport* (1998 to 2017), *Journal of Sport Behavior* (1990 to 2017), *Journal of Sport* & *Exercise Psychology* (1979 to 2017), *Journal of Sports Sciences* (1983 to 2017), *Psychology of Sport and Exercise* (2000 to 2017), *Medicine* & *Science in Sports* & *Exercise* (1969 to 2017), *Performance Enhancement* & *Health* (2012 to 2017), *Research Quarterly for Exercise and Sport* (2001 to 2017), *Scandinavian Journal of Medicine and Science in Sport* (1991 to 2017), *Sport, Exercise, and Performance Psychology* (2011 to 2017), *Substance Abuse Treatment, Prevention, and Policy* (2006 to 2016), and *The Sport Psychologist* (1987 to 2017). Finally, all reference lists of included papers were searched, a strategy that is sometimes referred to as Pearl Growing (Hartley, 1990). As previously mentioned, there were no date limits placed on any of the searches, so we included the start date in which the journals were first published. For example, the first edition of Addiction was published in 1903, so we searched this journal from 1903 until 2017.

### Eligibility criteria

**Figure 1:**
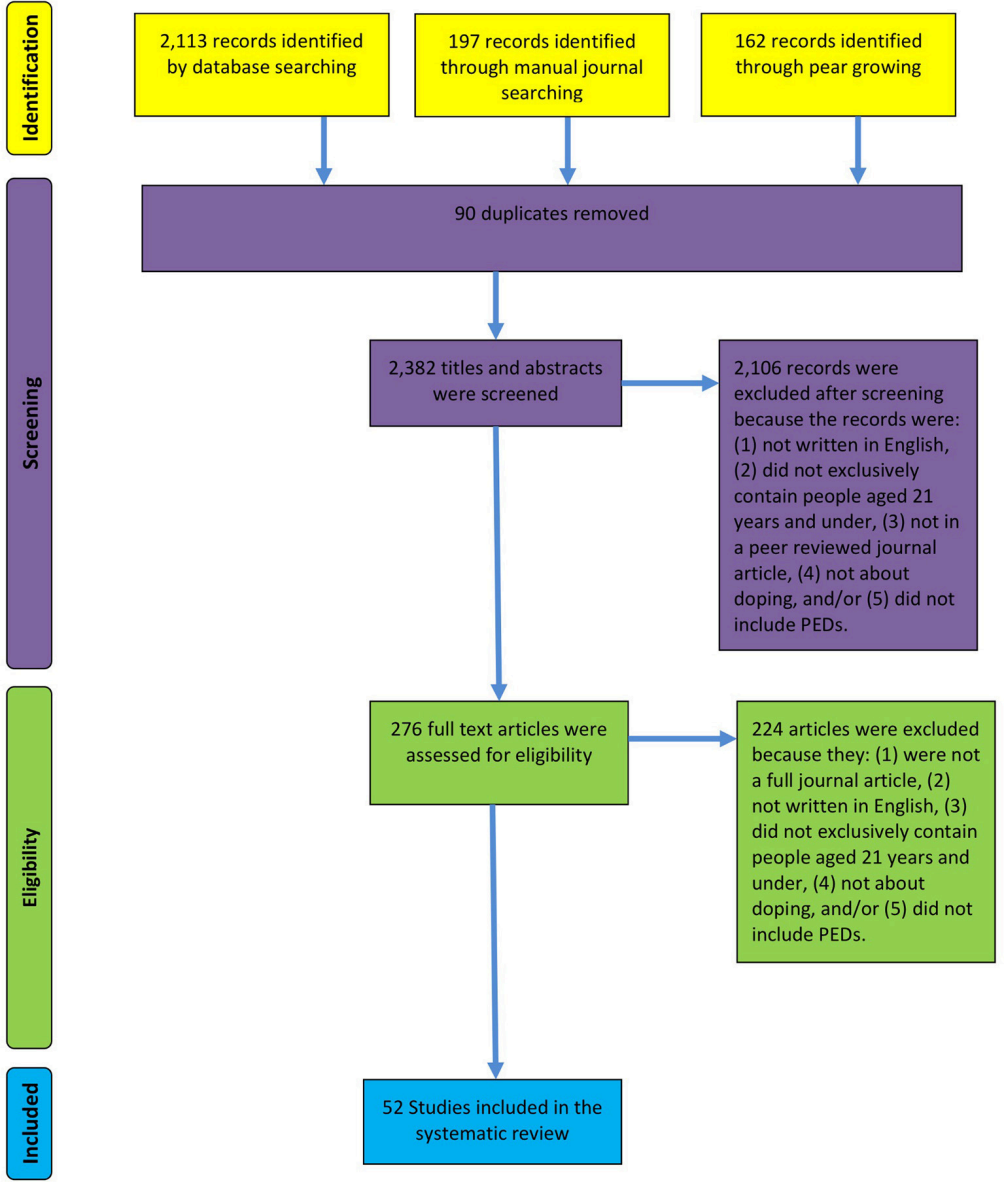
PRISMA flow diagram.

English language studies in peer-reviewed journals, which assessed the factors that influenced doping in relation to people aged up to 21 years were included. Samples that included young people in addition to those over 21 years old were excluded. For example, Thorlindsson and Halldorsson's (2010) paper was excluded. Even though the mean age of this sample was 17.7 years, the age of the sample ranged from 15 to 24 years. In total, 2,472 records, via the three different searches, were retrieved (see Figure 1). Ninety of these records were duplicates, so 2,382 titles and abstracts were screened. Based upon the eligibility criteria, 2,106 studies were excluded after reading the abstracts and titles. The full text of 276 papers was read, and then 224 papers were excluded because they did fulfill the inclusion criteria. Fifty-two studies fulfilled the study's inclusion criteria (see Figure 1 for a PRISMA flow diagram, which depicts the sequence of dataset selection and reasons for excluding articles).

These studies were subjected to an inductive content analysis procedure (Morehouse and Maykut, 2002). As such, similar predictors of doping were grouped together as themes. Each theme was assigned a descriptive label and a rule of inclusion was constructed for each theme. For example, one theme was descriptively labeled as “entourage.” The rule of inclusion for entourage was “other people that were associated with the athlete (e.g., parents, coaches, siblings, peers, or medical staff) and influenced whether a young person would dope or not.” Another theme was descriptively labeled as “sports participation.” The rule of inclusion was “participating in sport influenced whether or not an athlete would dope.” Eventually, all the findings were categorized into one of 9 themes that predicted doping. In order to assess the accuracy of the themes and rules of inclusion, the second and eighth authors read each theme and rule of inclusion, and discussions took place until there was total agreement.

### Assessment of methodological quality and risk of bias

An adapted version of the Cochrane Collaboration's Risk of Bias tool (Higgins et al., 2011) was used by the first author, following the guidance of Ntoumanis et al. (2014). This guide included a framework for assessing bias among experimental, cross-sectional, and longitudinal studies (see Table 1 note for the risk criteria). The Cochrane Collaboration's Risk of Bias tool provides an overall risk of bias of low, high, or unclear. Studies that scored low risk on all criteria were considered low risk, whereas studies that scored high risk on one criterion were considered high risk, and studies that scored unclear on one criterion were scored as unclear (see Table 1 for criteria scores for each study and Table 2 for overall risk bias evaluations). To assess the accuracy of the ratings by the first author, 25% of the papers were scored on the same criteria by the second author. There was a 95% consistency between independent assessments made by the first and second author. This was resolved after a discussion, and consensus was achieved for all items.

**Table 1:** Risk of bias summary.

	**1**	**2**	**3**	**4**	**5**	**6**	**7**	**8**	**9**	**10**	**11**	**12**	**13**	**14**	**15**
Barkoukis et al., 2015	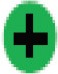	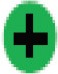	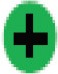	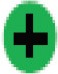	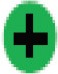	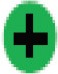	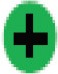								
Barkoukis et al., 2015	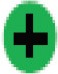	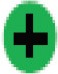	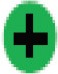	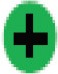	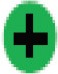	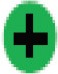	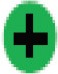								
Blank et al., 2015	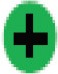	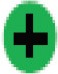	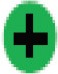	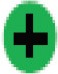	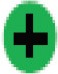	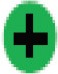	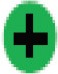								
Blank et al., 2016	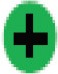	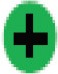	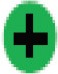	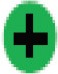	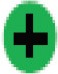	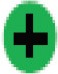	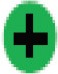								
Blashill et al., 2017	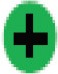	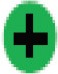	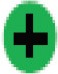	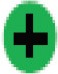	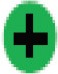	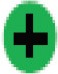	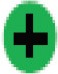								
Bloodworth et al., 2012	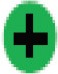	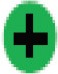	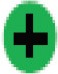	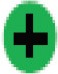	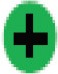	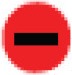	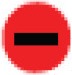								
Chan et al., 2015a (JSMS)	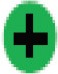	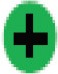	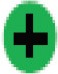	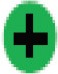	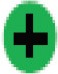	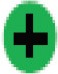	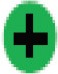								
Chan et al., 2015b (JSEP)	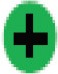	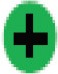	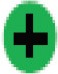	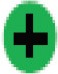	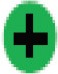	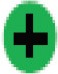	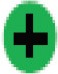								
Corbin et al., 1994	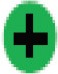	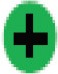	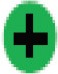	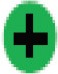	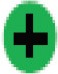	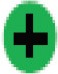	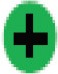								
Dodge and Clarke, 2015	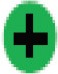	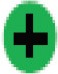	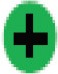	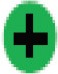	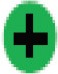	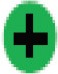	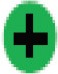								
Dodge and Jaccard, 2006	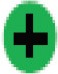	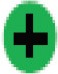	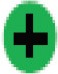	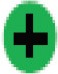	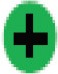	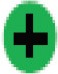	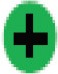								
Dodge and Jaccard, 2006	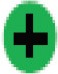	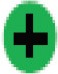	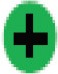	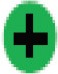	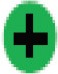	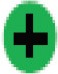	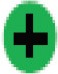								
Dunn and White, 2011	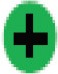	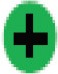	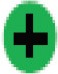	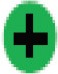	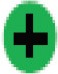	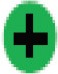	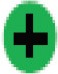								
DuRant et al., 1995	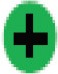	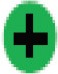	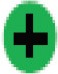	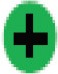	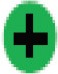	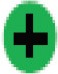	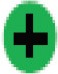								
Elkins et al., 2017	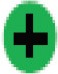	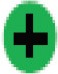	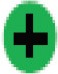	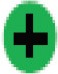	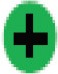	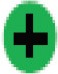	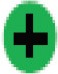								
Elliot et al., 2007	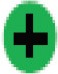	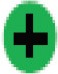	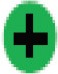	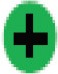	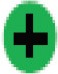	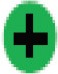	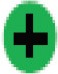								
Faigenbaum et al., 1998	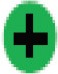	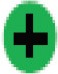	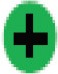	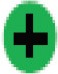	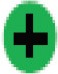	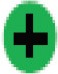	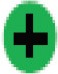								
Giraldi et al., 2015	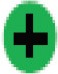	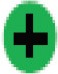	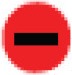	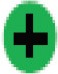	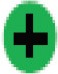	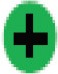	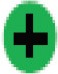								
Goldberg et al., 1991	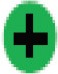	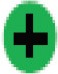	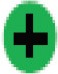	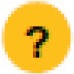	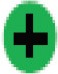	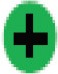	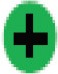	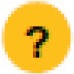	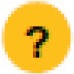						
Hoffman et al., 2008	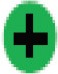	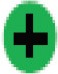	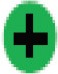	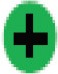	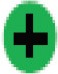	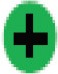	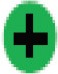								
Irving et al., 2002	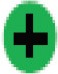	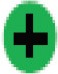	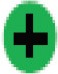	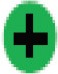	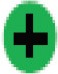	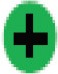	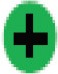								
Jampel et al., 2016	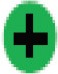	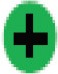	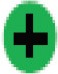	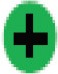	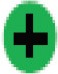	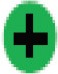	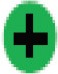								
Judge et al., 2012	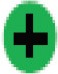	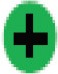	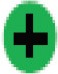	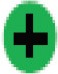	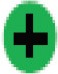	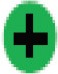	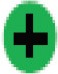								
Kindlundh et al., 1999	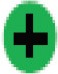	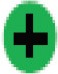	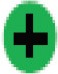	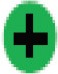	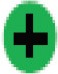	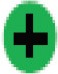	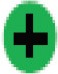								
Laure et al., 2004	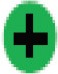	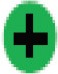	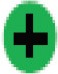	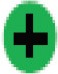	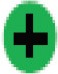	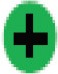	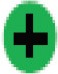								
Laure and Binsinger, 2007	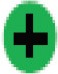	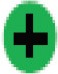	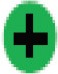	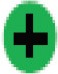	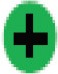	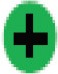	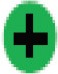	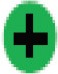	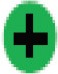						
Lazuras et al., 2015	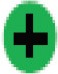	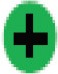	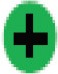	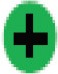	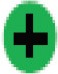	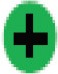	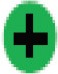								
Lucidi et al., 2004	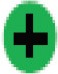	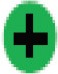	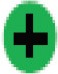	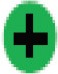	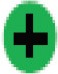	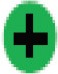	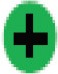								
Lucidi et al., 2008	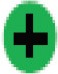	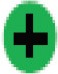	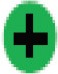	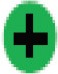	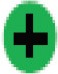	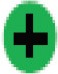	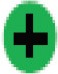	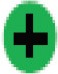	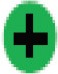						
Lucidi et al., 2013	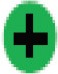	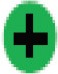	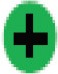	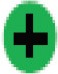	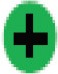	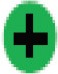	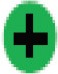	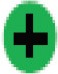	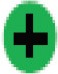						
Madigan et al., 2016	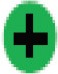	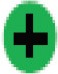	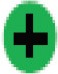	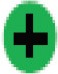	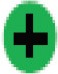	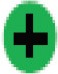	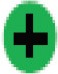								
Mallia et al., 2013	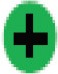	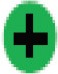	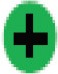	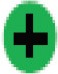	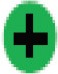	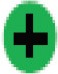	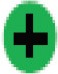								
Mallia et al., 2016	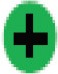	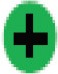	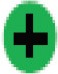	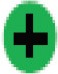	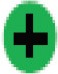	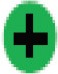	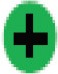								
Melia et al., 1996	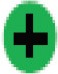	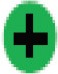	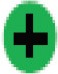	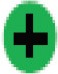	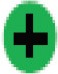	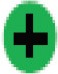	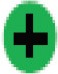								
Miller et al., 2002	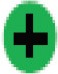	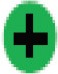	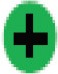	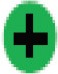	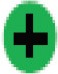	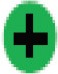	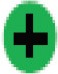								
Moston et al., 2015	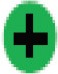	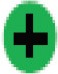	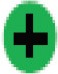	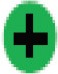	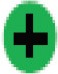	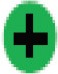	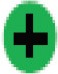								
Naylor et al., 2001	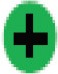	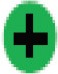	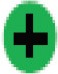	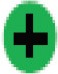	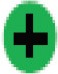	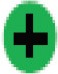	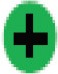								
Nicholls et al., 2015		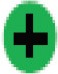	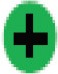		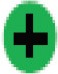										
Nilsson, 1995	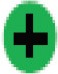	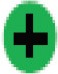	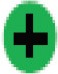	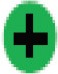	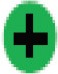	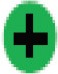	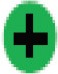								
Rees et al., 2008	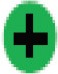	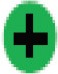	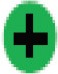	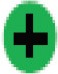	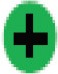	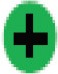	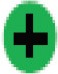								
Pedersen and Wichstrøm, 2001	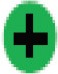	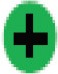	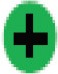	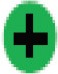	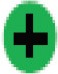	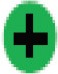	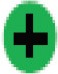								
Sagoe et al., 2016	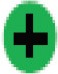	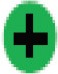	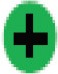	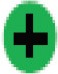	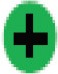	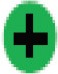	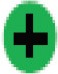								
Schirlin et al., 2009	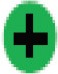	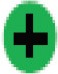	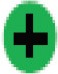	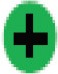	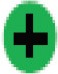	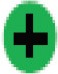	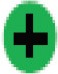			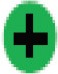	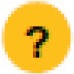	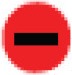	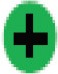	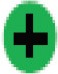	
Stilger and Yesalis, 1999	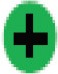	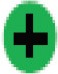	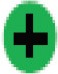	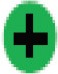	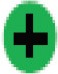	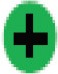	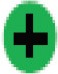								
Terney and McLain, 1990	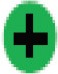	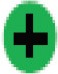	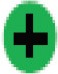	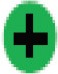	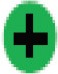	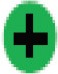	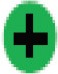								
vandenBerg et al., 2007	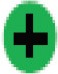	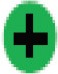	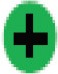	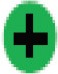	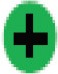	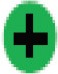	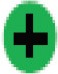	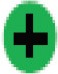	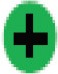						
Wanjek et al., 2007	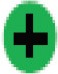	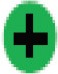	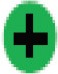	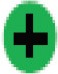	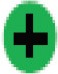	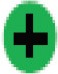	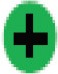								
Wichstrøm, 2006	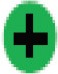	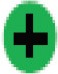	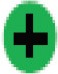	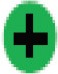	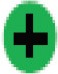	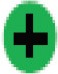	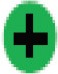	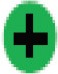	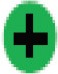						
Woolf et al., 2014	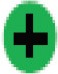	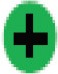	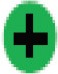	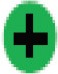	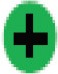	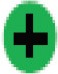	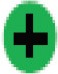								
Wroble et al., 2002	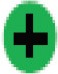	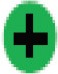	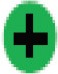	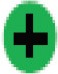	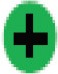	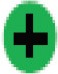	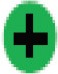								
Zelli et al., 2010a—(JCSP)	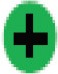	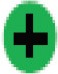	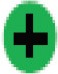	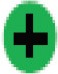	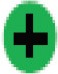	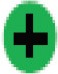	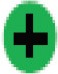	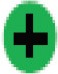	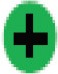						
Zelli et al., 2010b—(PSE)	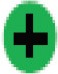	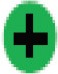	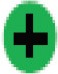	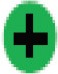	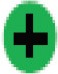	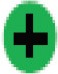	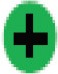	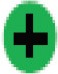	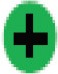						

*Risk of Bias 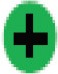, Low; 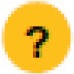, Unclear; 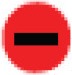, High. The risks of bias of studies included were assessed using the criteria below. Studies were assessed as having (a) no or low risk of bias, or (b) potential risk of bias. Criterion for all studies involved: Sampling (1. Participants are randomly selected, 2. Sample sizes are adequate, 3. Participants are representative of various demographic groups, 4. If some participants were excluded from the analyses, the exclusion is justified, 5. When group comparisons were made, participants were matched on other meaningful demographics, and 15. Other risks of bias), and measures (i.e., 6. Validated measures are used, or the authors have provided sufficient supportive information of the psychometric properties of the measures they devised and 7. Measures used were clearly defined and were appropriate). The criterion for studies that adopted a longitudinal or prospective design included: 8. Authors examined whether dropout is random, 9. Missing data were treated appropriately. Finally, the following criterion was used for experimental designs: 10. Allocation sequence generated to produce comparable groups. 11. Allocation was concealed, 12. Whether blinding was done and the effectiveness of it, 13. Outcome data for all outcomes were reported. Incomplete outcomes due to attrition and exclusions were addressed, and 14. No selective outcome reporting*.

**Table 2:** Study characteristics.

**Study**	**Participant Information**	**Instrumentation**	**Design**	**Risk of bias assessment**	**Main findings**
Barkoukis et al., 2015	309 adolescent Greek athletes, aged between 14 and 18 years old (*M* age = 16.64 years). This included 178 males and 131 females.	Questionnaires to assess beliefs about sporting success, current and past PEDs usage, attitudes, norms, situational temptation, and social desirability.	Cross-sectional	Low	Those who believed that deception strategies (i.e., cheating) were needed to be successful in sport were also more susceptible to doping. Athletes who possessed favorable doping attitudes and who felt others approved of them doping were more likely to take PEDs.
Barkoukis et al., 2015	650 athletes from Greece, who were aged between 14 and 20 years old (*M* age = 16.09 years). Gender not reported.	Questionnaires to assess doping intentions, attitudes, norms, and beliefs about NS use.	Cross-sectional	Low	NS users, who did not take PEDs, reported stronger intentions to dope, more favorable doping attitudes and beliefs, compared to non-supplement users.
Blank et al., 2015	883 parents of junior athletes.	Questionnaires to assess knowledge of PED, side effects of PEDs, and doping attitudes.	Cross-sectional	Low	Male parents reported greater knowledge of PEDs and their side effects than female parents. There were no significant gender differences in attitudes toward doping.
Blank et al., 2016	1,265 junior athletes from Australia, aged between 14 and 19 years. Mean age or gender not reported.	Questionnaires to assess doping susceptibility, well-being, goal orientation, confidence of success, performance motivation, depressive mood, anxiety, and locus of control.	Cross-sectional	Low	Low self-esteem, fear of failure, ego orientation, and a depressive mood were associated with the athletes who were the most susceptible to doping.
Blashill et al., 2017	6,248 14 to 18 year olds from the United states.	Questions to measure ethnicity, sexual orientation, and AAS use.	Cross-sectional	Low	Sexual minority boys were more likely to take AAS than heterosexuals. These differences were more pronounced among Black and Hispanic males.
Bloodworth et al., 2012	403 athletes aged between 12 and 21 years of age who were classified as being talented. 67% of the athletes were aged between 16 and 19 years.	Questions relating to the beliefs about the necessity of PEDs, body satisfaction and modification, and willingness of competitors to use PEDs.	Cross-sectional	High	Older athletes (17 to 20 years) with more than 5 years' experience agreed that PEDs were necessary in their sport. Of these, males (18%) displayed more favorable attitudes toward PEDs than females (10%). In turn, those who felt that taking PEDs was necessary in their sport were more likely to possess favorable attitudes toward using PEDs. Although many athletes reported that they would not take a magic (and undetectable PED; <10%) PED, 72.6% believed that others would use a magic PED if it were un-harmful, and 40% believed others would use a PED if it shortened one's life.
Chan et al., 2015a	410 elite or sub-elite athletes from Australia (*M* age = 17.7; male = 55.4%).	Behavioral regulation, self-regulation, and doping avoidance in relation to the theory of planned behavior.	Cross-sectional	Low	Athletes with an autonomous motivation for sport had an autonomous motivation for avoiding doping (i.e., doping was against personal values), and an attitude of doping avoidance. Alternatively, athletes with a controlled motivation had a controlled motivation for doping avoidance (e.g., wanting to avoid ban or gaining a poor reputation).
Chan et al., 2015b	410 elite or sub-elite athletes from Australia (*M* age = 17.7; male = 55.4%).	Trait self-control, doping attitudes, doping intentions, adherence to doping avoidant behaviors, and prevention of unintended doping behaviors. Participants also took part in a Lollipop decision-making task.	Cross-sectional	Low	Self-control was negatively associated with doping attitudes and intentions to use PEDs. Self-control was also associated with adherence and intention to avoid doping behaviors and a refusal to eat the unfamiliar lollipop presented in the decision-making task. Athletes with less self-control were more likely to have a favorable doping attitude and a stronger intention to dope, but reduced adherence to doping avoidance behaviors.
Corbin et al., 1994	1,690 US high-school athletes aged 13 to 17 years.	Questions to assess previous use of AAS, attitudes, and peer influence.	Cross-sectional	Low	2.4% of males and 1.1% of females reported using AAS. Males reported being able to obtain AAS more easily than females. 10% reported they would use AAS if they were guaranteed an Olympic medal.
Dodge and Clarke, 2015	244 students from the US, aged between 12 and 18 years (*M* age = 15.8 years), which comprised of 118 males, 121 females, 5 people who did not report gender.	Questionnaires to assess willingness to use AAS, behavioral intentions, amount of communication, and past substance use.	Cross-sectional	Low	Students engaged in little communication about AAS with their parents, although conversations about the positive impact of AAS on performance was associated with an increased willingness to use newly developed PEDs.
Dodge and Jaccard, 2006	Approximately 15,000 (exact number not provided) US males and females age 12 to 18 years.	Study created questions to assess steroid, supplement, physical activity, demographics, and substance use (e.g., binge drinking, use of drugs, and injection drugs).	Cross-sectional	Low	Males (2.7%) were more likely than females (0.4%) to use AAS, and these differences were greatest for those who played high-school sports. There was a positive relationship between legal dietary supplements and AAS use.
Dodge and Jaccard, 2008	241 adolescent athletes (*M* age = 15.8 years) from the US (154 males, 81 males, and 6 participants who did not report their gender).	Questionnaires to assess intentions, attitudes, social norms, substance use, and beliefs about doping.	Cross-sectional	Low	2.5% of the sample reported taking a PED. Attitudes and norms favoring abstinence from PEDs were the strongest predictors of intentions to take PEDs and legal supplements.
Dunn and White, 2011	22,830 Australian students aged between 12 and 17 years of age (*M* age = 14.5 years). 53% of the sample were females.	Questions to assess life time use of steroids, other illegal substances, and demographic variables.	Cross-sectional	Low	2.4% of the sample reported using AAS. The 12–15 years olds reported using AAS more than 16–17 year olds. Independent of age, factors associated with an increased use of AAS were being male, speaking a non-English language at home, not being at school the previous day, and rating one's educational ability as being below average. AAS use was also associated with using illegal substances.
DuRant et al., 1995	12,272 US high-school students aged 14 to 18 years old.	Study created questions to assess frequency of anabolic steroids, cigarettes, and other illegal drugs.	Cross-sectional	Low	Males (4.08%) were more likely than females to use anabolic steroids (1.2%). 16–18 year olds were more likely to take AAS than 15-year-olds. There were differences in relation to where athletes lived (i.e., higher prevalence of ASS use in south, than Northeast or West of US). Among males, there was a positive association between those who used had injected drugs, other illegal drugs, alcohol and AAS. Those who had participated in strength training were more likely to use AAS. ASS was not associated with level of performance. Among females, other drug use, followed by injected drugs were the strongest predictors of AAS.
Elkins et al., 2017	38,414 adolescents, aged between 14 and 18 years. 51% female.	Questions to assess demographics, AAS usage, and other drugs.	Cross-sectional	Low	2.6% of the sample reported using AAS within the year prior to the assessment. AAS were more common among male, older, and ethnic minority pupils.
Elliot et al., 2007	7,447 US female students aged between 14 and 18 years old.	AAS usage.	Cross-sectional	Low	5.3% of female students reported using AAS. Caucasian females were more likely to use AAS than Hispanic or African-American students. 14 and 15 year olds were more likely to report AAS than 18 year olds. AAS engaged in health harming behaviors such as drink driving, having more sexual partners, not wearing a seatbelt, being a passenger with a drink driver, or carrying a gun more than non AAS users. Athletes were less likely to use ASS than non-athletes.
Faigenbaum et al., 1998	965 US athletes (466 males, 499 females), aged between 9 and 13 years (*M* age = 11.4 years).	Questions to assess AAS usage, attitudes, and perceptions toward AAS.	Cross-sectional	Low	2.6% of males and 2.8% of females abused AAS. 58% of users compared to 31% of non-users reported that AAS made their muscles bigger. Users (31%) and non-users (11%) thought that AAS improved performance. Users (23%) knew someone their own age who was taking AAS, whereas only 9% of non-users did. 54% of users compared to 91% of non-users felt AAS were bad for them. 38% of users and 4% of non-users were asked to take AAS.
Giraldi et al., 2015	423 soccer players from Italy who were aged under 15 years (53%) or between 16-18 years old (47%), although the exact age of the 15 and under soccer players was not reported. 94.3% of the sample were male.	Questionnaires regarding attitudes and opinions regarding sports participation, reasons for winning, using PEDS enhance performance, opinions about self-image, in addition to views, knowledge, and behavior regarding doping, NS, and medical appointments for athletes.	Cross-sectional	High	50% of the sample was unaware of the health-related side effects that PEDs can cause. 6.5% of the males, but none of the females considered doping to enhance their performance.
Goldberg et al., 1991	192 high-school varsity American football teams from the US.	Study created questions to use of AAS and supplements, in addition to beliefs, opinions, and attitudes toward AAS.	Longitudinal (2 weeks between assessments).	High	Negative education programs did not increase athletes' beliefs of adverse effects of AAS, whereas those who received a balanced education program (i.e., positive and negative effects of AAS) were more aware of the adverse effects of AAS.
Hoffman et al., 2008	3,248 students from the 12 states in the US, aged between 13 and 18 years old. Sample comprised of 1,559 males and 1,689 females. Mean age not provided.	Questions relating to demographics, NS use, AAS use, attitudes and beliefs toward AAS, and main sources of education.	Cross-sectional	Low	1.2% of the sample abused AAS (2.4% of males and 0.8% of females). NS use was associated with the consumption of AAS, and this association was stronger among the male participants, who also reported using more NS. In particular, males who used supplements for increased strength or body mass, were the nutritional users who were most likely to use AS. Among males and females, supplements designed to reduce body mass or body fat were associated with students using AAS. The use of AAS was higher among 18 year olds than 13 year olds. AAS was significantly higher among 14 to 18-year-old males than females, with 6% of 17-18-year-old males abusing AAS. Teachers and parents were the main source of information regarding supplements and AAS, although parents were less important by the time the students were 17 to 18 years old. As parents became less important, older students relied more on friends, coaches, trainers, and the internet, with older males reporting strength and conditioners as being more important than females.
Irving et al., 2002	4,746 middle and high-school athletes from the US.	Study created questions to assess AAS use, demographics, psychological constructs, and behaviors.	Cross-sectional	Low	AAS was more common in males than females. Non-Caucasians and middle school students (in comparison with high-school students) were more likely to use AAS. AAS among males was linked to depressed mood, less knowledge of health and poor attitude to health, sports where weight and shape were important, disordered eating, and substance use. The pattern for females was less consistent than males, although the results were similar.
Jampel et al., 2016	6,000 US males aged between 14 and 18 years old (*M* age = 16).	Questions to assess ASS use, weight, height, sports participation, weight change attempts, and asthma history.	Cross-sectional	Low	Males who perceived that they were under weight or over weight were more at risk of using AAS throughout their lifetime.
Judge et al., 2012	98 track and field athletes from the United States, who were aged between 12 and 19 years old (*M* age for males = 15.2 years, *M* age for females = 15.8 years). Sample comprised of 46 males and 52 females.	Questionnaire to assess attitudes, subjective (including both injunctive and descriptive) norms, perceived behavioral control, moral convictions, history of doping, and doping intentions.	Cross-sectional	Low	There were no gender differences in regards to doping intentions. The use of PEDs was predicted by attitude strength, injunctive norms, and moral conviction.
Kindlundh et al., 1999	2,742 Sdish students, comprising of 1,592 16 to 17 year olds, and 1,150 18 to 19 year olds.	Questions about PEDs, behavioral activities, and lifestyle.	Cross-sectional	Low	Those involved in strength training, tobacco use, large alcohol intake, living alone, and playing truant at least once per week were linked to the participants consuming PEDs. Some illegal substances (e.g., cannabis oil, LSD, opioids, and amphetamine) were positively associated with AAS use.
Laure et al., 2004	1,459 males and female high-school athletes from France.	4-page questionnaire pack to assess drug use, beliefs about doping (including psychological factors).	Cross-sectional	Low	4% of athletes reported using PEDs, which were generally supplied by friends or health professionals. PEDs users perceived that PEDs posed less of a health threat than non-users. Those who used PEDs were less anxious and more confident than non-users, but also perceived that they were not happy or healthy.
Laure and Binsinger, 2007	3,564 students aged 11-12 years old from France started the study and 2,199 of these completed the second assessment 4 years later.	PED use, sports participation, self-esteem, and trait anxiety.	Longitudinal (assessments every 6 months for 4 years).	Low	1.2% reported taking PEDs as an 11 or 12-year-old, which rose to 3% by the time the students were 14 or 15 years old and there was also an increase in the frequency of use. PED use increased with training hours and level of competition, and was higher among males than females. Some PED users are also more anxious and report lower self-esteem than non-users.
Lazuras et al., 2015	650 Greek athletes, aged 14 to 20 years old (*M* age = 16.09 years). 68.3% were males.	Questionnaires regarding social desirability, achievement goals, motivation regulation, sportspersonship, social cognitive variables (e.g., attitudes, norms, and self-efficacy), and anticipated regret.	Cross-sectional	Low	4.2% reported they committed a doping violation. The model assessed variables that accounted for 57.2% of doping intentions variance. Social cognitive factors and anticipated regret directly influenced intentions to dope. Indeed, anticipated regret predicted doping intentions over and above the other variables. Current or past use of PEDs did not predict future doping intentions.
Lucidi et al., 2004	952 Italian students aged between 14 to 20 years old (*M* age = 16.48 years). 50.1% of the sample were males.	Questionnaire pack that contained three sections: (1) sporting activities over last 3 months, (2) use of NS and PEDs, and (3) behavioral intentions, attitudes, subjective norms, and perceived behavioral control.	Cross-sectional	Low	3.1% of the sampled reported using a PED. Those who participated in competitive sport or recreational sport were more likely to use PEDs than sedentary people. Males used more PEDs than females, across all PED types (e.g., AAS, growth hormones, and stimulants etc.). Attitudes were the strongest predictor of intentions to use PEDs, followed by subjective norms (medium), moral disengagement, and then perceived behavioral control (small). Past use of ergogenic aids predicted intentions to use PEDs.
Lucidi et al., 2008	1,232 Italian high-school students assigned to psychometric condition (variables assessed 3 weeks apart; 51% females, *M* age = 17 years) or longitudinal condition (*n* = 762; 51% females, *M* age = 16.8 years).	Study created questionnaire to assess psychological factors (e.g., behavioral intentions, attitudes, subjective norms, and perceived behavioral control, and doping-specific self-regulatory efficacy) that predict doping on two separate occasions, either 2 weeks or 3 months apart. Those in the longitudinal group also reported the PEDs consumed within 3 months after completing the Time 1 assessments, at Time 2.	Longitudinal (3 months between assessments)	Low	Males portrayed more favorable attitudes toward doping, greater approval from others, more eager to justify using PEDs, and possessed stronger intentions to use PEDs than females. Females expressed a stronger capacity to overcome personal or interpersonal pressure to use PEDs. 2.1% of the longitudinal group reported using a PED in the previous 3 months and more males reported doping during this time, than females. Intentions to take PEDs were linked to attitudes, others' approval, doping, and the belief that PEDs were morally justified. Participants who reported a stronger moral disengagement or intentions to use PEDs at Time 1, were more likely to dope in the intervening 3 months. Being able to resist pressure from others and stronger perceptions of self-control were negatively associated with PED use. The prevalence of PEDs correlated significantly and positively with nutritional supplementation.
Lucidi et al., 2013	1,975 Italian high-school students, aged between 13 and 18 years old (*M* age = 16.3 years).	Questionnaires assessed moral disengagement, attitudes toward doping, self-regulatory efficacy to resist social pressure, social norms relating to doping approval, doping intentions, and motivational orientation at Time 1, and use of PEDs and supplements at Time 2 (3 months later).	Longitudinal (3 months between assessments).	Low	Athletes used different ways of justifying doping use. They denied the effects of PEDs or displaced responsibility. Moral disengagement was also associated with doping intentions and PEDs abuse.
Madigan et al., 2016	129 male athletes from the United Kingdom, aged between 16 and 19 years old (*M* age = 17.3 years).	Questionnaire to measure perfectionism and attitudes toward doping.	Cross-sectional	Low	Parental pressure was positively associated with favorable attitudes toward doping. The authors reported a negative relationship between perfectionism strivings and positive doping attitudes. after controlling for all overlapping variables.
Mallia et al., 2013	3,400 Italian high-school students (50.9% males, *M* age = 16.5 years, *SD* = 1.6).	Participants completed a 5-point scale to assess doping attitudes and reported previous use of doping in the 3 months prior to data collection.	Cross-section	Low	Males, older adolescents within the sample, and athletes reported using PEDs than their counterparts (e.g., females, younger adolescents, and those not involved in sport). Further, males, older participants, and athletes were more likely to report a favorable attitude toward the use of PEDs.
Mallia et al., 2016	Study 2 contained 414 adolescent athletes (*M* age = 16.69 years), and Study 3 contained 749 team athletes (*M* age participated in Study 3) from Germany, Greece, and Italy.	Interviews and Questionnaires to assess self-regulatory efficacy in teams, team collective efficacy, and moral disengagement in relation to doping.	Cross-sectional	Low	Self-regulatory efficacy in team contexts was negatively associated with intentions to dope during the next season, whereas as team moral disengagement was positively associated with intentions to dope during the next season.
Melia et al., 1996	16,169 students from Canada aged 11 to 18.	Questions relating to PED usage and attitudes toward PEDs.	Cross-sectional	Low	2.8% used AAS in the year prior to the survey, with 29.4% of these athletes injecting AAS. Of these, 29.2% shared needles. AAS was used mainly used to alter body composition rather than enhancing performance, whereas other PEDs were used to enhance sports performance.
Miller et al., 2002	16 175 US students who were aged between 14 and 19 years.	AAS usage.	Cross-sectional	Low	There were no significant gender differences. Those with parents with poorer educational achievements took more AAS. AAS users took more risks in other areas of their life such as with alcohol and in their sexual behaviors, and scored higher on suicidal risk. Girls who used AAS scored higher on suicide risk than males. Male and female users reported similar scores on alcohol and substance abuse. Female athletes were less likely to use AAS than non-athlete females.
Moston et al., 2015	312 athletes (aged between 12 and 17 years old) who were either elite or non-athletes.	Questionnaires assessed moral functioning and participants reported perceived incidence of doping violations.	Cross-sectional	Low	Participants estimated that 28.4% of athletes doped, with those in athletics, weightlifting, and cycling thought to commit the most doping offences. Elite athletes perceived that 11.26% of their competitors doped. 12 to 13 year olds and 16-17 years old estimated a higher level of doping violations that the 14 to 15 year olds. There were no gender differences.
Naylor et al., 2001	1,515 high-school students from the US (grades 912).	Questions relating to drug use and knowledge of rules.	Cross-sectional	Low	There were no differences between athletes and non-athletes in AAS use. 32% of athletes were unaware of anti-doping rules regarding the use of recreational drugs during the season.
Nicholls et al., 2015	11 coaches from four countries, who coached adolescent athletes in athletics, basketball, kayaking, racquetball, rowing, rugby league, or rugby union.	Semi-structured interviews.	Cross-sectional	Low	Coaches reported that attitudes toward doping among adolescents were influenced by perceptions of threat of being caught and the perceived benefits of PEDs. Additionally, those with low morality and low self-esteem were also thought to have a favorable attitude toward PEDs. Friends and family's opinions were also perceived to be important in influence doping attitudes. Age or maturation, level of participation, pressure experienced, country of residence, and ethnicity were reported as factors that influenced doping attitudes.
Nilsson, 1995	1,383 students aged between 14 and 19 years (688 males and 695 females).	Questions to assess demographics and AAS usage.	Cross-sectional	Low	5.8% of males used AAS. 10% of 15-16 year olds used AAS and half of these injected AAS as a liquid.
Pedersen and Wichstrøm, 2001	10,828 Norwegian students aged between 14 and 17 years old.	Questions to assess use of PEDs, demographics, parental control leisure time activities, and substance abuse.	Cross-sectional	Low	2.3% of males reported using PEDs, compared to 1.3% of females. Using PEDs was associated with alcohol exposure from parents and poor monitoring, in addition to using gymnasiums, using smokeless tobacco, and alcohol abuse. There was strong associated between PEDs and illegal drugs (e.g., amphetamines, MDMA, and heroin).
Rees et al., 2008	495 US high-school students aged 14 to 18 years.	Questions to assess attitudes, sensation seeking, and supplement use.	Cross-sectional.	Low	Supplement users reported a stronger intention to take PEDs than non-NS users.
Sagoe et al., 2016	1,334 Norwegian 18-year-olds (females 58.7%).	Questionnaires to measure aggression, AAS intent alcohol disorders, anxiety, and depression.	Cross-sectional	Low	Those who were more aggressive reported a stronger intent to use AAS in the future, in comparison with their less aggressive counterparts.
Schirlin et al., 2009	97 adolescents (54.6% male), aged between 11-15 years).	Reaction times on Stroop emotion task (doping, cheating, and control words). Scales on physical self-perception, physical self-worth, physical condition, sport competence, body attractiveness, and physical strength.	Experimental	Potential	Reaction times were longer after doping words than either cheating or control words, inferring that attentional resources allocated to doping are already present by the time a person reaches adolescence. Further, low self-esteem may be a risk factor for doping, as those in the low self-esteem group demonstrated larger carry-over effects (i.e., significant slowdown of response times due to difficulty in disengaging attention from another word or stimuli) than those in the high self-esteem group.
Stilger and Yesalis, 1999	873 varsity American football players from urban, suburban, and rural high schools.	Questions relating to knowledge and attitudes toward AAS, and usage, availability, reasons to use, dosage, and method of using AAS.	Cross-sectional	Low	6.3% were previous or current abusers of AAS. Ethnic minority players (e.g., Hispanic or Asians; 11.2%) were twice as likely to use AAS than Caucasian (6.5%) players. AAS use increased with playing experience and was linked to specific positions (e.g., linebackers). Other players, doctors and coaches were the most reported source for obtaining AAS. The age when players first used AAS was: <10 years, *n =* 10), 11–12 years (*n* = 10), 13–15 years (*n* = 24), and 16 to 18 years (*n* = 22). AAS were used to enhance performance, followed by improving appearance, and to keep up with other competitors. Of those who did not take AAS, health concerns, against beliefs, and cost were the main reasons.
Terney and McLain, 1990	2,113 high-school students aged between 14 and 18 years old.	Questions relating to attitudes, usage, knowledge, and availability of AAS, and how these are perceived by others.	Cross-sectional	Low	There was a higher incidence of AAS use among athletes (5.5%) in comparison with non-athletes (2.4%). Males, American footballers, and wrestlers were more likely to take AAS than females or those involved in other sports. 2% of athletes reported that their coach or another member of staff recommended the use of AAS. 9% reported that they would consider using AAS to gain a college scholarship.
vandenBerg et al., 2007	2,516 students from the US, aged 11 to 18 years of age.	Questions to assess AAS use, demographics, eating activity, weight, and related variables.	Longitudinal (5 years between assessments).	Low	1.5% of the sample abused AAS, although the numbers of students taking AAS remained stable and thus did not increase with age. Male AAS users wanted to weigh more, whereas female AAS users reported higher BMIs, were unsatisfied with their weight, had poorer knowledge nutrition, and less concern for their health.
Wanjek et al., 2007	2,319 adolescents from Germany.		Cross-sectional	Low	15.1% of athletes reported using a PED in the previous year. Non-athletes, recreational athletes, and competitive athletes generally scored poorly on a tested that assessed knowledge of doping. Older athletes and those with a more favorable attitude toward taking PEDs were more like to dope. No significant differences between non-athletes, recreational athletes, and competitive athletes for AAS, but non-athletes took stimulants more than competitive or recreational level athletes.
Wichstrøm, 2006	2,924 Norwegian high-school students (aged 15–19 years).	Questions completed on AAS use, involvement in power sports, appearance and eating problems, problematic behavior, and emotional problems. All questions completed at Time 1 (baseline), Time 2 (2 years later), and Time 3 (5 years after baseline).	Longitudinal (5 years between assessments).	Low	1.9% reported using AAS at some point during the study. Male, an involved in power sports, problematic behaviors (e.g., misconduct, cannabis abuse, alcohol abuse, and sexual debut before 15th birthday) predicted AAS use at Time 3.
Woolf et al., 2014	404 male adolescent athletes, aged 14 to 19 years old (*M* age = 16.06 years), predominantly Caucasian.	Questionnaires to measure intentions, descriptive norms, injunctive norms, outcome expectations, and control variables.	Cross-sectional	Low	Athletes believed that AAS would benefit their performance, which was linked to intentions. Intentions to use AAS were influenced by injunctive norms. Athletes who believed that professional athletes used ASS more than their team mates. Athletes who believed that their team mates used AAS they had a stronger intention to use AAS than if they believed other professional athletes were using AAS. As such, perceptions of what team mates do may be more important than perceptions of professional athletes in predicting doping intentions.
Wroble et al., 2002	1,553 athletes aged between 10 and 14 years from the US, with 1,079 males and 474 females. The age of the participants was 10 (*n =* 248), 11 (*n =* 394), 12 (*n =* 484), 13 (*n =* 274), 14 (*n =* 199), or 15 (*n =* 32) years of age.	15 questions that measured demographics, prevalence of AAS, knowledge of side effects, attitudes, and where to obtain AAS from.	Cross-sectional	Low	0.9% of males and 0.2% of females reported taking AAS. Of these users, 27% did so for performance, 18% to improve personal appearance, and 18% for body building. 18% took AAS due to peer pressure. 90% of athletes did not feel they needed to take AAS to be successful. The most prominent sources of information were books/magazines, parents, and coaches.
Zelli et al., 2010a	1,022 Italian students (balanced sample, around 16 years of age) completed Time 1 and 864 students (*M* age = 16.5 years, 51% female) completed Time 2, 4 months later.	Questionnaires regarding drive for muscularity, drive for thinness, doping attitudes, and doping intentions.	Longitudinal (4 months between assessments)	Low	Drive for thinness and muscularity were positively associated with intentions to dope. Drive for muscularity (but not thinness) exerted this effect through positive doping attitudes.
Zelli et al., 2010b	1,022 Italian students (balanced sample, around 16 years of age) completed Time 1 and 864 students (*M* age = 16.5 years, 51% female) completed Time 2, 4 months later.	Questionnaires assessed doping attitudes, subjective norms, behavioral control, self-regulatory efficacy, moral disengagement, and prior substance use.	Longitudinal (4–5 months between assessments)	Low	47% of the variance in doping intentions was predicted by favorable attitudes toward doping, strong views that significant others would approve, a tendency to justify using PEDs, and lower confidence in one's ability to resist social pressure. Moral disengagement was associated with favorable attitudes toward doping and intentions to dope. Doping behavior remained stable over the 4 months, intentions at Time 1 predicted the use of PEDs at Time 2.

## Results

### Study characteristics

Fifty-two studies explored factors that influenced doping among young people aged 21-years-old and under (see Table 2). These 52 studies included 187,288 participants, with most participants aged between 14 and 18 years. There were notable exceptions that included either younger participants or older participants. For example, Faigenbaum et al. (1998) included participants aged between 9 and 13 years, and Laure and Binsinger (2007) assessed participants aged between 11 and 12 years old. Conversely, Lazuras et al. (2015) included participants up to the age of 20 years old, and Bloodworth et al. (2012) included participants who were aged between 12 and 21 years of age. One study assessed parents' (Blank et al., 2015) and another study (Nicholls et al., 2015) assessed coaches' opinions regarding factors that influence doping among adolescent athletes. The number of participants involved in these studies ranged from 11 (Nicholls et al., 2015) to 16,175 (Miller et al., 2002). Forty-two studies were cross-sectional, 9 were longitudinal, and one was experimental. The amount of time between the first and final assessment in the longitudinal studies ranged from 2 weeks (Goldberg et al., 1991) to 5 years (Wichstrøm, 2006). Most studies included males and females, but five studies recruited males (Goldberg et al., 1991; Stilger and Yesalis, 1999; Woolf et al., 2014; Jampel et al., 2016; Madigan et al., 2016), and two studies recruited females only (Laure et al., 2004; Elliot et al., 2007). Young people from Australia, France, Germany, Greece, Italy, Norway, Sweden, the United Kingdom, and the United States were represented in the studies included in this systematic review.

### Factors that predict doping among young people

Based on the analysis of the data, nine factors that predicted doping among young athletes: gender; age; sport participation; sport type; psychological variables; entourage; ethnicity; nutritional supplements (NS); and health harming.

### Gender

Thirteen studies reported an incidence of doping among young males and females, and one study explored gender differences in relation to the parents of adolescent athletes (Blank et al., 2015). The prevalence of doping among young people in the different samples ranged from 0.9 to 6% for males, and between 0.2 and 5.3% for females. Eight studies specifically compared the prevalence of doping among males and females (e.g., Corbin et al., 1994; Pedersen and Wichstrøm, 2001; Wroble et al., 2002; Dodge and Jaccard, 2006; Hoffman et al., 2008; Dunn and White, 2011; Mallia et al., 2013; Elkins et al., 2017) and reported a higher incidence of doping among young males than young females. One study reported a higher incidence of doping among females than males (e.g., Faigenbaum et al., 1998), and one study found no differences (e.g., Miller et al., 2002). Giraldi et al. (2015) compared perceptions of males and females regarding the effects of doping on performance, with 6.5% of males, but none of the females believing that PEDs benefit sports performance, although there were only 24 females in this study. Nevertheless, gendered beliefs may explain why 6% of 17 to 18-year-old male students in Hoffman et al. (2008) study reported using AAS. In contrast to the studies that explored gender differences among young people, Blank et al. (2015) examined whether parents of adolescent athletes reported different attitudes toward doping and whether their knowledge of PEDs was different. There were no differences between mothers and fathers in relation to doping attitudes, but fathers possessed more knowledge about PEDs than mothers. Overall, the weight of evidence suggested that there was a greater incidence of doping among young males than young females.

### Age

Eleven studies explored age as a variable that influenced doping or perceptions of doping among young people. For example, Laure and Binsinger (2007) examined the prevalence of doping among a sample of 3,564 French students, aged 11 to 12-years-old. Researchers assessed the participants every 6 months over 4 years, via questionnaires, which culminated in the participants reporting their doping behavior on 8 occasions. The number of young people using PEDs increased with age: 1.2% reported a doping violation at the start of the study, increasing to 3% of the sample 4 years later. Similarly, Wanjek et al. (2007) reported that older adolescents from Germany were more likely to dope than younger adolescents, as did Hoffman et al. (2008), Elkins et al. (2017), and Mallia et al. (2013). One explanation regarding the trend of doping increasing with age is that older adolescents feel greater pressure to be successful in sport (e.g., win competitions or secure professional contracts) or to increase their muscle mass (Eppright et al., 1997). Bloodworth et al. (2012) reported that the oldest athletes in their sample of 12 to 21 year olds, with over 5 years of training experience, felt that it was necessary to take PEDs to be successful. However, another longitudinal study examined the prevalence of AAS among a sample 5 years apart, and reported that the prevalence usage remained stable (vandenBerg et al., 2007). Although some studies (e.g., Laure and Binsinger, 2007; Wanjek et al., 2007; Hoffman et al., 2008) found a clear relationship between doping prevalence and age, Moston et al. (2015) explored the extent to which young athletes estimated the prevalence of doping and did not find a linear pattern. They reported that 12- to 13- and 16- to 17-year-olds believed that more young athletes were doping than 14- to 15-year-olds. The estimation of doping prevalence did not necessarily increase with age. It should be noted, however, that Moston and colleagues did not actually explore the prevalence of doping. In support of Moston's finding that there was not a linear pattern between doping and age, Elliot et al. (2007) found that 14- and 15-year-old females were more likely to report using AAS than 18-year olds. Similarly, Dunn and White (2011) reported that 12 to 15 year olds were more likely to misuse AAS than 16 to 17 year olds. Stilger and Yesalis (1999) examined the age in which high-school American football players first started using AAS. The authors reported that 15.2% of the sample first abused AAS before their 10th birthday and 15.2% also used AAS for the first time between the age of 11 and 12 years of age. The average age that the sample first used AAS was when they were 14 years old. The evidence regarding doping and age among young people is equivocal, because some studies reported that older adolescents were more likely to take PEDs than younger people, whereas other studies reported a higher prevalence of doping among younger groups of adolescents than older age groups.

### Sports participation

Five studies compared the prevalence of doping among young people who played sport and those who did not partake in competitive sport. Elliot et al. (2007), Naylor et al. (2001), and Wanjek et al. (2007) reported no differences between athletes and non-athletes regarding the use of AAS. Wanjek et al., however, found that non-athletes were more likely to take stimulants than recreational or competitive athletes. In contrast to the findings regarding AAS abuse, Naylor et al. (2001) reported a higher incidence of AAS abuse among athletes compared to non-athletes, with 5.5% of athletes and 2.4% of non-athletes using AAS, and Mallia et al. (2013) reported a higher incidence of doping among athletes in comparison to non-athletes. Similarly, Lucidi et al. (2004) found a higher incidence of doping among competitive and recreational athletes in comparison to non-athletes. Overall, the evidence is mixed, as some studies reported a higher incidence of PEDs among athletes than non-athletes, whereas other studies reported no differences.

### Sport and activity type

Six studies identified differences in the prevalence of doping among young people in relation to the sport or activity type. Involvement in strength-based sports or activities was associated with higher incidence of doping. For example, Wichstrøm (2006) reported an involvement in sports predicted who misused AAS. Further, DuRant et al. (1995), Kindlundh et al. (1999), and Pedersen and Wichstrøm (2001) reported a higher incidence of doping among young people involved in strength training or who attend a gymnasium on a regular basis. Terney and McLain (1990) revealed that young people aged between 14 and 18 years old who played American football or wrestled reported a higher instance of doping compared to those who played other sports, and Irving et al. (2002) reported that doping was more prevalent in sports where athletes perceive that their weight and body shape is important. Although, Stilger and Yesalis (1999) did not examine the relationship between doping prevalence and sport or activity type, they explored differences in doping among American football players across different playing positions. They found that 59% of AAS users played as lineman, linebacker, or a defensive end, which are the positions that require strong and powerful athletes. Participating in sports where strength and body shape is an important determinant of successful performance predicted doping among young people.

### Psychological variables

Twenty-one studies identified 22 psychological factors that were related to doping (see Table 3). Psychological constructs such as aggression (Sagoe et al., 2016), anticipated regret (e.g., Lazuras et al., 2015), attitudes (e.g., Zelli et al., 2010a), deception strategies (e.g., Barkoukis et al., 2015), depressive mood (e.g., Irving et al., 2002), drive for muscularity and thinness (e.g., Zelli et al., 2010a), ego-orientation (e.g., Blank et al., 2016), fear of failure (e.g., Blank et al., 2016), intentions (e.g., Lucidi et al., 2004), moral disengagement (e.g., Mallia et al., 2016), social or injunctive norms, resisting social pressure (Zelli et al., 2010b), suicide risk (Miller et al., 2002), and susceptibility (e.g., Barkoukis et al., 2015) were positively associated with doping. Conversely, psychological constructs such as happiness (Laure et al., 2004), self-control (Chan et al., 2015b), self-esteem (Nicholls et al., 2015), moral conviction (Judge et al., 2012), and perfectionist strivings (Madigan et al., 2016) were negatively associated with doping. Different psychological variables acted as a protective mechanism against doping (e.g., self-esteem, resisting social pressure, and perfectionist strivings) or were associated with higher incidence of doping (e.g., drive for muscularity, anticipated regret, or aggression).

**Table 3:** Psychological factors that predict doping among young people.

**Psychological construct**	**Study in which the psychological constructs appeared**
Aggression	Sagoe et al., 2016
Anxiety	Laure and Binsinger, 2007
Anticipated Regret	Lazuras et al., 2015
Attitudes Toward Doping	Barkoukis et al., 2015
	Bloodworth et al., 2012
	Dodge and Jaccard, 2008
	Hoffman et al., 2008
	Judge et al., 2012
	Lucidi et al., 2004, 2008, 2013
	Mallia et al., 2013
	Nicholls et al., 2015
	Zelli et al., 2010b
Deception Strategies	Barkoukis et al., 2015
Depressive Mood	Blank et al., 2016
	Irving et al., 2002
Drive for Muscularity	Zelli et al., 2010a
Drive for Thinness	Zelli et al., 2010a
Ego-orientation	Blank et al., 2016
Fear of Failure	Blank et al., 2016
Happiness	Laure et al., 2004
Intentions	Dodge and Jaccard, 2008
	Lazuras et al., 2015
	Lucidi et al., 2004
	Woolf et al., 2014
Motivation	Chan et al., 2015a
Moral Conviction	Judge et al., 2012
Moral Disengagement	Lucidi et al., 2004, 2008
	Mallia et al., 2016
	Zelli et al., 2010b
Perfectionism	Madigan et al., 2016
Resisting Social Pressure	Zelli et al., 2010b
Self-control	Chan et al., 2015b
	Lucidi et al., 2004, 2008
Self-esteem	Blank et al., 2016
	Laure and Binsinger, 2007
	Nicholls et al., 2015
Self-regulatory Efficacy	Mallia et al., 2016
Norms	Barkoukis et al., 2015
	Dodge and Jaccard, 2008
	Judge et al., 2012
	Lucidi et al., 2008
	Nicholls et al., 2015
	Woolf et al., 2014
	Zelli et al., 2010b
Susceptibility toward Doping	Barkoukis et al., 2015 Blank et al., 2016

### Entourage

Nine studies reported how an athlete's entourage (i.e., parents, coaches, friends, physiotherapists, doctors, or strength and conditioning coaches) influenced doping. Terney and McLain (1990) found that 2% of athletes reported a coach had previously recommended that they take AAS, with coaches, doctors, and players being the most frequently cited members of an athlete's entourage to obtain AAS (Stilger and Yesalis, 1999). Coaches in Nicholls' et al. (2015) study believed that susceptible athletes would take PEDs if their coach asked them to, which aligns to Madigan et al.'s (2016) finding that pressure from coaches was associated with favorable doping attitudes. Coaches may possess a strong influence over young athletes, because some athletes may view coaches as one of their main source of information (Wroble et al., 2002). Parents also influenced the prevalence of doping among young people too. For example, children of parents with low educational achievements were more likely to take PEDs, as were those who were exposed to alcohol more and received less monitoring by their parents (Pedersen and Wichstrøm, 2001). The friends or peer groups of young people were also found to influence doping. Wroble et al. (2002) reported that 18% of AAS users took this substance due to pressure from their friends. Indeed, the study by Laure et al. (2004) revealed that PEDs were mainly supplied by either friends or health professionals.

Teachers and parents were the main source of information regarding supplements and AAS, although parents were less important by the time the students were 17 to 18 years old (Hoffman et al., 2008). As parents' influence declined, older students relied more on friends, coaches, trainers, and the internet, with older males reporting strength and conditioning coaches as being more important. An athlete's entourage influenced whether an athlete would dope or decide against doping, because coaches, parents and friends could act as a preventive or facilitative mechanism toward doping.

### Ethnicity

Five studies explored the relationship between ethnicity and doping. Elliot et al.'s (2007) sample of 7,447 US female students revealed that Caucasian students were more likely to take AAS than either Hispanic or African-American students. Conversely, in Stilger and Yesalis's (1999) sample of 873 male high-school American Football players, those of a Hispanic or Asian descent were nearly twice more likely to abuse AAS than Caucasian players. Indeed, 11.2% of Hispanic or Asian players doped, in comparison with just 6.5% of Caucasian players. Further, Elkins et al. (2017) reported that AAS use was more common in African-American and Hispanic students. Blashill et al. (2017) examined AAS among sexual minority and heterosexual males, and found that across Black, Hispanic, and Caucasian adolescents there was a higher incidence of AAS use than among other ethnicities. However, these differences were more pronounced among Black and Hispanic males than Caucasians. Four of the coaches in Nicholls' et al. (2015) qualitative study believed that some young Caucasian rugby players in New Zealand would be more tempted to dope because some of their competitors from other ethnic backgrounds (e.g., Polynesians) are “predominately a lot larger than your average Caucasian young man” (p. 98). This coach believed that many coaches select players based on size across young age groups, so there would be pressure for Caucasian players to take PEDs. The findings regarding ethnicity and doping were equivocal, so there may be other factors that contribute to doping rather than just ethnicity exclusively, such as education background, socio-economic status, or the functional demands of a sport (e.g., necessity to be strong, powerful, or lean).

### Nutritional supplements

Six studies reported the relationship between NS use (e.g., amino acid, creatine, and protein) and doping. All six studies (e.g., Lucidi et al., 2004, 2008; Dodge and Jaccard, 2006; Hoffman et al., 2008; Rees et al., 2008; Barkoukis et al., 2015) reported a positive relationship between NS use and the prevalence of PEDs or intentions to use PEDs. The relationship between NS and PEDs was stronger for male participants than female students. Young males reported were more frequent users of NS than young females (Hoffman et al., 2008). Further, males who used supplements for increased strength or body mass, were the most likely to also take AAS. The use of supplements designed to reduce body mass or body fat were reported among both males and females were also associated with students using AAS. Barkoukis et al. (2015) compared the attitudes of NS and non-NS users who did not dope. Those who consumed NS reported a stronger intention to dope, more favorable doping attitudes and beliefs about PEDs, in comparison with non-supplement users. Using nutritional supplements was associated with young people abusing PEDs or going on to take PEDs later in their life.

### Health harming behaviors

Seven studies explored the relationship between health harming behaviors and the prevalence of PEDs. A variety of health harming behaviors were positively associated with young people abusing PEDs. These included alcohol abuse (e.g., DuRant et al., 1995; Pedersen and Wichstrøm, 2001; Miller et al., 2002; Wichstrøm, 2006; Dunn and White, 2011), illegal substance, such as cannabis or heroin (e.g., DuRant et al., 1995; Kindlundh et al., 1999; Pedersen and Wichstrøm, 2001; Wichstrøm, 2006), drink driving, having more sexual partners, not wearing a seatbelt, and being a passenger with a drink driver (Elliot et al., 2007). Young people with less concern for their health, and thus engaged in a variety of different behaviors that may harm their health were more likely to dope.

## Discussion

The purpose of this review was to provide an overview and analysis of the factors that predicted doping among young people. Fifty-two studies fulfilled the inclusion criteria. These studies yielded nine factors that predicted doping among young people. These were gender, age, sports participation, sport type, psychological variables, an athlete's entourage, ethnicity, NS, and health harming behaviors. Twenty-two different psychological variables were associated with doping among young people. Although these studies were vital in predicting doping, they did not fully explain why young people doped. Researchers could attempt to explain why these 9 factors are associated with doping, because this information may be used to enhance the efficacy of education programs. Young males were more likely to use PEDs than young females, as five studies reported a higher prevalence of doping in males than females, whereas only one study reported a higher incidence of females, and one study found no significant difference. Although these studies examined gender differences, there were few attempts to explain why males are more likely to take PEDs than females. This goes beyond the scope of this systematic review, but it would be interesting to examine the factors that contributed to these findings. One possible explanation relates to the perceptions of PEDs, as Giraldi et al. (2015) reported that males were more likely to perceive that PEDs benefitted performance in comparison with females. This could be one factor that explains gender differences in relation to PEDs. There could also be other factors, too, such as those that contribute toward gender differences. This could be due to different levels of involvement between young males and females in strength training or participation in sports associated with increased use of PEDs, as these were associated with increased PEDs use (e.g., DuRant et al., 1995; Kindlundh et al., 1999; Pedersen and Wichstrøm, 2001; Wichstrøm, 2006), or more males using NS than females (e.g., Hoffman et al., 2008). Further, as an athlete's entourage can impact on doping behavior and attitudes (e.g., Nicholls et al., 2015; Madigan et al., 2016), it is possible that coaches or peers may exert a different influence on males in comparison with females, which then could influence gender differences in relation to doping. Additionally, males tend to use different members of their entourage than females, such as strength and conditioning coaches (e.g., Hoffman et al.). Finally, there could be differences in key psychological variables that predict doping (e.g., drive for muscularity) among males and females. Clearly, this is speculation, but most studies reported a higher incidence of doping among males in comparison to females and future research endeavors could explore factors that contribute to these gender differences in doping. This will provide a greater insight into the reasons why both males and females take PEDs, which could inform the development of gender specific education. Overall, it appeared that PED abuse increased as young people matured through childhood and adolescence (Stilger and Yesalis, 1999; Laure and Binsinger, 2007), although this might not be true for specific PEDs, such as AAS, because the use of AAS was stable (e.g., vandenBerg et al., 2007). It is a cause for concern that some young people have taken PEDs before their 10th birthday (Stilger and Yesalis, 1999), and the average age in which Stilger and Yesalis reported young people first take AAS was 14-years-old. These findings indicate the need for education regarding PEDs beginning during childhood, and certainly by the time a young person reaches adolescents. This is because attitudes and values are formed during middle childhood (Döring et al., 2015; Cieciuch et al., 2016; Kjellström et al., 2017) and studies in this systematic review reported a positive association between attitudes and doping use or intentions to use PEDs (e.g., Zelli et al., 2010b; Judge et al., 2012; Barkoukis et al., 2015). If young people are exposed to anti-doping education in their late teens, it could be too late as some people will already be PED users and their attitudes will be formed, which makes changing attitudes more difficult (Hartan and Latané, 1997). As such, bespoke anti-doping interventions for child and adolescent athletes that utilize a variety of engaging platforms (such as face-to-face sessions and mobile applications) are urgently required. In recent years, the emphasis of scholarly activity has somewhat shifted toward the psychological factors that predicted doping rather than just assessing prevalence or demographic factors associated with doping. Indeed, over 85% of the studies that explored psychological factors and doping among young people were published in the last 10 years. So far, researchers have identified 22 different psychological factors that were associated with doping among young people. It is likely that other psychological factors will emerge, given the growth of funding opportunities in doping research. Exploring the prevalence of these psychological factors can be a method of identifying young people who are at risk of doping without specifically measuring doping intentions. If risk factors are identified early in a young person's sporting careers, there is the potential that these people could receive education before their first experimentation with PEDs, which could ultimately reduce the numbers of young people who take PEDs. Proactive, rather than re-active, education or psychological interventions could be valuable in reducing the prevalence of certain psychological constructs (e.g., favorable attitudes toward doping, drive for thinness and/or muscularity, fear of failure, and ego-orientation), whilst enhancing protective psychological constructs such as self-esteem, self-control, and pleasant emotions such as happiness. Another factor that may predict doping among young people is personality. Personality was cited as a factor that influences doping in two theoretical frameworks, the Sport Drug Control Model (Donovan et al., 2002) and the Sport Drug Control Model for Adolescent Athletes (Nicholls et al., 2015). Although scholars are yet to test the relationship between personality and doping specifically among young people, a recent study by Nicholls et al. (2017b) found a significant relationship between attitudes toward doping and the Dark Triad of personality, namely Machiavellianism, psychopathy, and narcissism. It should be noted, however, that Machiavellianism and psychopathy explained 29% of the variance doping attitudes toward doping, but narcissism did not independently predict doping attitudes. This study was conducted with adult athletes, but future scholarly activity could explore personality constellations (Paulhus and Williams, 2002) and the Big Five personality traits (McCrae and Costa, 2003) with young people. Even though scholars are yet to test the relationship between personality constellations and doping, researchers did explore trait versions of psychological constructs such as perfectionism (Madigan et al., 2016) and trait anxiety (Laure and Binsinger, 2007). Although Madigan et al. found an association between perfection and doping attitudes, more contemporary research raised questions over the validity of their findings (Nicholls et al., 2017a). Madigan and colleagues used the Performance Enhancement Scale (PEAS; Petróczi and Aidman, 2009) to assess doping attitudes among junior athletes. However, Nicholls et al. (2017a) reported that the PEAS demonstrated a poor model fit for athletes aged 17-years and under. To verify Madigan's finding, researchers could use a doping attitude questionnaire that is validated with young people. This could be problematic, because many studies developed their own scale to assess doping attitudes (e.g., Lucidi et al., 2004, 2008, 2013; Zelli et al., 2010b) without validating these questionnaires. As such, there is a need for a questionnaire specifically designed and validated to assess doping attitudes among young athletes from several countries so that scholars around the world have an accurate scale at their disposal. If they could use such a questionnaire, it would make comparisons between studies more accurate and promote cross-cultural research. The relationship between NS use and doping is not a new finding, although has worrying implications for the future. Indeed, Lucidi et al. (2004) first identified a relationship between doping and NS use among young people, which was confirmed in subsequent studies (e.g., Dodge and Jaccard, 2006; Hoffman et al., 2008; Lucidi et al., 2008). Interestingly, Hoffman et al. (2008) explored the reasons for consuming NS and AAS abuse. They found that males who took NS for strength or body mass gains were the most likely to use AAS, whereas males and females who took NS for either weight or fat loss were likely to take AAS. As such, identifying the reasons why young people take NS is another mechanism for governing bodies, schools, or NADOs identifying those who are at greatest risk of abusing AAS without specifically asking about their future intentions. With the NS industry set to increase exponentially over the next few years, with conservative estimates of it being worth over $60 billion by 2021 (Lariviere, 2013), there could also be an increase in the number of young people taking dietary supplements. This in turn may then lead to more people taking PEDs, as those who take supplements tend to have relatively strong intentions to take PEDs (Barkoukis et al., 2015). This is a concern for the future, so the use of supplements and PEDs needs to be carefully monitored among young people over the next few years. Although 9 predictors of doping emerged in this systematic review, it is plausible that other factors could predict doping among young people. With the exception of Miller et al. (2002), who examined the relationship between parental educational attainment and the use of PEDs among their children, researchers are yet to clearly establish whether a young person's educational attainment status predicts PED abuse. Another factor that could predict doping is a person's socio-economic status. This is because Origer et al. (2014) reported that education attainment and socio-economic status both predicted fatal overdoes from opioids and cocaine. It would be useful to identify whether education achievement and socio-economic status predicted doping among young people, because this would help policy makers and national governing bodies help identify those that may be at risk of doping, if education attainment and socio-economic status predict the use of PEDs.

A limitation of this systematic review is that most studies (78.8%) were cross-sectional. This represents a limitation of the doping literature, because only an association and not causation can be inferred from cross-sectional research. It should be noted, however, that cross-sectional research is useful at assessing the prevalence of a behavior (e.g., doping) among a specific population (Sedgwick, 2014). As commented upon previously, scholars have used a variety of different self-report questions and questionnaires to assess doping prevalence or factors such as attitudes toward doping without validating these questionnaires. It is plausible however, that the young people may have underestimated the extent to which they reported whether they consumed PEDs or not honestly answered questions about attitudes toward doping honestly, although many scholars asked participants to complete questionnaires anonymously. An alternative approach to assessing doping attitudes questionnaires is to assess implicit attitudes. Brand et al. (2014) used a picture based technique and assessed the reaction times of participants. Although, it should be noted that this technique was validated using the 17-item PEAS 9 Petróczi and Aidman, 2009), and Nicholls et al. (2017a) reported a poor model fit for the 17-item PEAS among adults and adolescents. As such, it could be argued that Brand et al.'s method may require additional validation with a more robust psychometric scale. Another limitation is that 51% of the studies in this systematic review focused exclusively on AAS, and thus did not measure other PEDs. As such, some young people who were using or had a history of using other PEDs would be undetected in nearly half of the studies. Further, this may have contributed to equivocal findings regarding the relationship between doping and age. Laure and Binsinger (2007) assessed the prevalence of PEDs over a four-year period and found an increase as people matured, whereas vandenBerg et al. (2007) reported no increase in the use of AAS. Future research could address a much broader range of PEDs rather than just AAS to ascertain an accurate measurement of doping among young people. Researchers could also conduct research among participants from different countries within the same studies. Although there are studies featuring athletes from different countries, due to scholars using different scales, it is difficult to compare psychological variables, as factors that might predict doping and thus whether it impacts on doping behavior. Scholarly activity could compare athletes from different countries to see if there are any differences, which would be helpful in generating education programs, specific to the needs of athletes. Finally, although widely recognized search techniques were employed to identify papers, it is still possible that relevant articles were missed. This is because some articles may not appear in a search engine result, due to the keywords selected or might not be referenced in the journal articles cited in the systematic review, and would therefore be missed by the search engine and pearl growing. It is also plausible that some relevant articles could be published in journals that were not manually searched, despite searching 27 relevant different journals.

Given that attitudes can form in childhood and early adolescence (Döring et al., 2015; Cieciuch et al., 2016; Kjellström et al., 2017), it is important that children and young adolescents are exposed to anti-doping messages through education programs. Although scholars are yet to examine the effectiveness of anti-doping education programs among children, the Athletes Training Learning and to Avoid Steroids (ATLAS; Goldberg et al., 1996a,b, 2000; Goldberg and Elliot, 2005) and Athletes Targeting Healthy Exercise and Nutrition Alternatives (ATHENA; Goldberg and Elliot, 2005; Elliot et al., 2008) were tested via randomized controlled trials (RCTs) among adolescents. Ntoumanis et al. (2014) meta-analysis reported a small, but significant effect of the ATLAS and ATHENA programs on doping intentions. Unfortunately, these education programs did not influence doping behaviors. The limited impact of these programs may be due to ATLAS and ATHENA not focusing exclusively on anti-doping education (Ntoumanis et al., 2014). As such, there is a need for specific anti-doping programs, which are specifically designed for young people.

In conclusion, young males are more likely to dope than young females and the prevalence and frequency of PEDs appears to increase with age during adolescence, although the number of young people taking AAS may remain stable. The type of sport in which an individual performs also predicts doping, as do psychological variables such as attitudes, self-esteem, and ego-orientation. People surrounding a young person (e.g., parents, coaches, peers) also impact upon doping, as do other behaviors such as using NS or the use of illegal drugs. These findings can be used to help identify young people at risk of doping, and many of the psychological factors can be manipulated through psychological interventions, which may help reduce the prevalence of PEDs among young people. Our findings can also inform pro-sport educational programs. Finally, as some people may take PEDs before their 10th birthday, young people should be exposed to anti-doping education before the onset of adolescence.

## Author contributions

All authors listed have made a substantial, direct and intellectual contribution to the work, and approved it for publication.

### Conflict of interest statement

The authors declare that the research was conducted in the absence of any commercial or financial relationships that could be construed as a potential conflict of interest.

## References

[B1] Backhouse S. McKenna J. Robinson S. Atkin A. (2007). Attitudes, Behaviors, Knowledge and Education - Drugs in Sport: Past, Present and Future. Research report submitted to the World Anti-Doping Agency, Canada.

[B2] Barkoukis V. Lazuras L. Lucidi F. Tsorbatzoudis H. (2015). Nutritional supplement and doping use in sport: possible underlying social cognitive processes. Scand. J. Med. Sci. Sport. 25, e582–e588. 10.1111/sms.12377 25556707

[B4] Bird S. R. Goebel C. Burke L. M. Greaves R. F. (2016). Doping in sport and exercise: anabolic, ergongenic, health, and clinical issues. Annal. Clin. Biochem. 53, 196–221. 10.1177/0004563215609952 26384361

[B5] Blank C. Leighfried V. Schaiter R. Fürhapter C. Müller D. Schobersberger W. (2015). Doping in sports: knowledge and attitudes among parents of Austrian junior athletes. Scand. J. Med. Sci. Sports 25, 116–124. 10.1111/sms.12168 24372621

[B6] Blank C. Schobersberger W. Leichtfried V. Duschek S. (2016). Health psychological constructs as predictors of doping susceptibility in adolescent athletes. Asian J. Sports. Med. 7:e35024. 10.5812/asjsm.35024 28144408 PMC5256272

[B7] Blashill A. J. Calzo J. P. Griffiths S. Murray S. B. (2017). Anabolic steroid misuse among US adolescent boys: disparities by sexual orientation and race/ethnicity. Am. J. Pub. Health 107, 319–321. 10.2105/AJPH.2016.303566 27997246 PMC5227934

[B8] Bloodworth A. J. Petróczi A. Bailey R. Pearce G. McNamee M. J. (2012). Doping and supplementation: the attitudes of talented young athletes. Scand. J. Med. Sci. Sport 22, 293–301. 10.1111/j.1600-0838.2010.01239.x 20973831

[B9] Brand R. Heck P. Ziegler M. (2014). Illegal performance enhancing drugs and doping in sport: a picture-based brief implicit association test for measuring athletes' attitudes. Subs. Abuse Treat. Prev. Policy 9:7. 10.1186/1747-597X-9-7 24479865 PMC3937137

[B10] Chan D. K. C. Dimmock J. A. Donovan R. J. Hardcastle S. Lentillon-Kaestner V. Hagger M. S. (2015a). Self-determined motivation in sport predicts anti-doping motivation and intention: a perspective from the trans-contextual model. J. Sci. Med. Sport 18, 315–322. 10.1016/j.jsams.2014.04.001 24793786

[B11] Chan D. K. C. Lentillon-Kaestner V. Dimmock J. A. Donovan R. J. Keatley D. A. Hardcastle S.. (2015b). Self-control, self-regulation, and doping in sport: a test of the strength-energy model. J. Sport. Exerc. Psych. 37, 199–206. 10.1123/jsep.2014-0250 25996110

[B12] Cieciuch J. Davidov E. Algesheimer R. (2016). The stability and change of value structure and priorities in childhood: a longitudinal study. Soc. Dev. 3, 503–527. 10.1111/sode.12147

[B13] Commission of the European Communities (2007). White Paper on Sport (2007). Available online at: http://eur-lex.europa.eu/legal-content/EN/TXT/?uri=CELEX:52007DC0391

[B14] Corbin C. B. Feyrer-Melk S. A. Phelps C. Lewis L. (1994). Anabolic steroids: a study of high school athletes. Ped. Exerc. Sci. 6, 149–158. 10.1123/pes.6.2.149

[B15] Dodge T. Jaccard J. J. (2008). Is abstinence an alternative? Predicting adolescent athletes' intentions to use performance enhancing substances. J. Health. Psychol. 13, 703–711. 10.1177/1359105307082460 18519443

[B16] Dodge T. L. Clarke P. (2015). Influence of parent–adolescent communication about anabolic steroids on adolescent athletes' willingness to try performance-enhancing substances. Subs. Use Misuse 50, 1307–1315. 10.3109/10826084.2014.998239 25629954

[B17] Dodge T. L. Jaccard J. J. (2006). The effect of high school sports participation on the use of performance-enhancing substances in young adulthood. J. Adolesc. Health 39, 367–373. 10.1016/j.jadohealth.2005.12.025 16919798

[B18] Donovan R. J. Egger G. Kapernick V. Mendoza J. (2002). A conceptual framework for achieving performance enhancing drug compliance in sport. Sport Med. 32, 269–284. 10.2165/00007256-200232040-00005 11929355

[B19] Döring A. K. Schwartz S. H. Cieciuch J. Groenen P. J. Glatzel V. Harasimczuk J.. (2015). Cross-cultural evidence of value structures and priorities in childhood. Brit. J. Psych. 106, 675–699. 10.1111/bjop.12116 25581067

[B20] Dunn M. White V. (2011). The epidemiology of anabolic–androgenic steroid use among Australian secondary school students. J. Sci. Med. Sport 14, 10–14. 10.1016/j.jsams.2010.05.004 20619732

[B21] DuRant R. H. Escobedo L. G. Heath G. W. (1995). Anabolic-steroid use, strength training, and multiple drug use among adolescents in the United States. Pediatrics 1, 25–28. 7596717

[B22] Elkins R. L. King K. Nabors L. Vidourek R. (2017). School and parent factors associated with steroid use among adolescents. J. School Health 87, 159–166. 10.1111/josh.12482 28147454

[B23] Elliot D. L. Cheong J. Moe E. L. Goldberg L. (2007). Cross-sectional study of female students reporting anabolic steroid use. Arch. Pediatr. Adolesc. Med. 161, 572–577. 10.1001/archpedi.161.6.572 17548762

[B24] Elliot D. L. Goldberg L. Moe E. L. DeFrancesco C. A. Durham M. B. McGinnis W. (2008). Long-term outcomes of the ATHENA (Athletes Targeting Health Exercise & Nutrition Alternatives) program for female high school athletes. J. Alcohol Drug Ed. 52, 73–92. PMC2598770 19081833

[B25] Eppright T. D. Sanfacon J. A. Beck N. C. Bradley J. S. (1997). Sport psychiatry in childhood and adolescence: an overview. Child. Psychiatry Hum. Dev. 28, 71–88. 10.1023/A:1025189118334 9494234

[B26] ESPAD (2011). ESPAD Report 2011: Substance Use Among Students in 36 European Countries. Available online at: http://www.espad.org/uploads/espad_reports/2011/the_2011_espad_report_full_2012_10_29.pdf

[B27] ESPAD (2015). ESPAD Report 2015: Results from the European School Survey Project on Alcohol and other Drugs. Available online at: http://www.espad.org/sites/espad.org/files/ESPAD_report_2015.pdf

[B28] Faigenbaum A. D. Zaichkowsky L. D. Gardner D. E. (1998). Anabolic steroid use by male and female middle school students. Pediatrics 101:e6. 10.1542/peds.101.5.e6 9565439

[B29] Giraldi G. Unim B. Masala D. Miccoli S. La Torre G. (2015). Knowledge, attitudes and behaviors on doping and supplements in young football players in Italy. Pub. Health 129, 1007–1009. 10.1016/j.puhe.2015.05.008 26119988

[B30] Goldberg L. Elliot D. Clarke G. N. MacKinnon D. P. Moe E. Zoref L.. (1996a). The adolescents training and learning to avoid steroids (ATLAS) prevention program: background and results of a model intervention. Archive. Ped. Adoles. Med. 150, 713–721. 10.1001/archpedi.1996.02170320059010 8673196

[B31] Goldberg L. Elliot D. L. (2005). Preventing substance use among high school athletes: the ATLAS and ATHENA programs. J. Appl. School Psych. 21, 63–87. 10.1300/J370v21n02_05

[B32] Goldberg L. Elliot D. L. Clarke G. N. MacKinnon D. P. Zoref L. Moe E. L. (1996b). The ATLAS (Adolescents Training and Learning to Avoid Steroids) program: effects of a multi-dimensional anabolic steroid prevention intervention. J. Am. Med. Assoc. 276, 1555–1562. 10.1001/jama.1996.03540190027025 8918852

[B33] Goldberg L. MacKinnon D. P. Elliot D. L. Moe E. L. Clarke G. Cheong J. (2000). Preventing drug use and promoting health behaviors among adolescent athletes: results of the ATLAS program. Archive. Ped. Adoles. Med. 154, 332–338. 10.1001/archpedi.154.4.332 10768668

[B34] Goldberg L. R. Bents E. Bosworth L. Trevsian Elliot D. L. (1991). Anabolic steroid education and adolescents: do scare tactics work? Pediatrics 87, 283–286. 2000267

[B35] Hartan H. C. Latané B. (1997). Social influence and adolescent life style attitudes. J. Res. Adoles 7, 197–220. 10.1207/s15327795jra0702_5

[B36] Hartley R. J. (1990). Online Searching: Principles and Practice. London: Bowker-Saur.

[B37] Higgins J. P. T. Altman D. G. Sterne J. A. C. (2011). Assessing risk of bias in included studies. in Cochrane Handbook for Systematic Reviews of Interventions, eds Higgins J. P. T. Green S. (The Cochrane Collaboration). Available online at: www.handbook.cochrane.org

[B38] Hoffman J. R. Faigenbaum A. D. Ratamess N. A. Ross R. Kang J. Tennenbaum G. (2008). Nutritional supplementation and anabolic steroid use in adolescents. Med. Sci. Sports. Exerc. 40, 15–24. 10.1249/mss.0b013e31815a5181 18091024

[B39] Irving L. M. Wall M. Neumark-Sztrainer D. Story M. (2002). Steroid use among adolescents: Findings from Project EAT. J. Adolesc. Health 30, 243–252. 10.1016/S1054-139X(01)00414-1 11927236

[B40] Jampel J. D. Murray S. B. Griffiths S. Blashill A. J. (2016). Self-perceived weight and anabolic steroid misuse among US adolescent boys. J. Adoles. Health 58, 397–402. 10.1016/j.jadohealth.2015.10.003 26598061 PMC4856064

[B41] Judge L. W. Bellar D. Petersen J. Lutz R. Gilreath E. Simon L. (2012). The attitudes and perceptions of adolescent track and field athletes toward PED use. Perform. Enhance. Health 1, 75–82. 10.1016/j.peh.2012.04.002

[B42] Kindlundh A. M. S. Isacsson D. G. L. Berglund L. Nyberg F. (1999). Factors associated with adolescent use of doping agents: anabolic-androgenic steroids. Addiction 94, 543–553. 10.1046/j.1360-0443.1999.9445439.x 10605850

[B43] Kjellström S. Sjölander P. Almers E. McCall M. E. (2017). Value systems among adolescents: novel method for assessing level of ego-development. Scand. J. Psych. 58, 150–157. 10.1111/sjop.12356 28252192

[B44] Lariviere D. (2013). Nutritional Supplements Flexing Muscles as Growth Industry. Forbes. Available online at: http://www.forbes.com/sites/davidlariviere/2013/04/18/nutritional-supplements-flexing-their-muscles-as-growth-industry/#57cec0708845

[B45] Laure P. Binsinger C. (2007). Doping prevalence among preadolescent athletes: a 4-year follow-up. Brit. J. Sports. Med. 41, 660–663. 10.1136/bjsm.2007.035733 17473000 PMC2465173

[B46] Laure P. Lecerf T. Friser A. Binsinger C. (2004). Drugs, recreational drug use and attitudes towards doping of high school athletes. Int. J. Sport. Med. 25, 133–138. 10.1055/s-2004-819946 14986197

[B47] Lazuras L. Barkoukis V. Tsorbatzoudis H. (2015). Toward an integrative model of doping use: an empirical study with adolescent athletes. J. Sport. Psych. 37, 37–50. 10.1123/jsep.2013-0232 25730890

[B48] Lindqvist A.-S. Moberg T. Ehrnborg C. Eriksson B. O. Fahlke C. Rosén T. (2013). Increased mortality rate and suicide in Swedish former elite male athletes in power sports. Scand. J. Med. Sci. Sport. 24, 1000–1005. 10.1111/sms.12122 24033718

[B49] Lucidi F. C. Grano L. Leone C. Lombardo Pesce C. (2004). Determinants of the intention to use doping substances: An empirical contribution in a sample of Italian adolescents. Int. J. Sport. Psych. 35, 133–148.

[B50] Lucidi F. Zelli A. Mallia L. (2013). The contribution of moral disengagement to adolescents' use of doping substances. Int. J. Sport. Psych. 44, 331–350.

[B51] Lucidi F. Zelli A. Mallia L. Grano C. Russo P. M. Violani C. (2008). The social-cognitive mechanisms regulating adolescents' use of doping substances. J. Sport. Sci. 26, 447–456. 10.1080/02640410701579370 18274942

[B52] Madigan D. J. Stoeber J. Passfield L. (2016). Perfectionism and attitudes towards doping in junior athletes. J. Sport. Sci. 34, 700–706. 10.1080/02640414.2015.1068441 26177255

[B53] Mallia L. Lazuras L. Barkoukis V. Brand R. Baumgarten F. Tsorbatzoudis H. (2016). Doping use in sport teams: the development and validation of measures of team-based efficacy beliefs and moral disengagement from a cross-national perspective. Psych. Sport. Exerc. 25, 78–88. 10.1016/j.psychsport.2016.04.005

[B54] Mallia L. Lucidi F. Zelli A. Violani C. (2013). Doping attitudes and the use of legal and illegal performance-enhancing substances among Italian students. J. Child Adolesc. Subs. Abuse 22, 179–190. 10.1080/1067828X.2012.733579

[B55] McCrae R. R. Costa P. T. (2003). Personality in adulthood: A five factor theory perspective. New York, NY: Guilford Press 10.4324/9780203428412

[B56] Melia P. Pipe A. Greenberg L. (1996). The use of anabolic androgenic steroids by Canadian students. Clin. J. Sport. Med. 6, 9–14. 10.1097/00042752-199601000-00004 8925377

[B57] Miller K. E. G. M. Barnes D. Sabo M. J. Melnick Farrell M. P. (2002). A comparison of health risk behavior in adolescent users of anabolic androgenic steroids, by gender and athlete status. Socio. Sport. J. 19, 385–402. 10.1123/ssj.19.4.385

[B58] Morehouse R. Maykut P. (2002). Beginning Qualitative Research: A Philosophical and Practical Guide (Teachers' Library). Washington, DC: Falmer Press.

[B59] Moston S. Engelberg T. Skinner J. (2015). Self-fulfilling prophecy and the future of doping. Psych. Sport. Exerc. 16, 201–207. 10.1016/j.psychsport.2014.02.004

[B60] Naylor A. H. D. Gardner Zaichkowsky L. (2001). Drug use patterns among high school athletes and non-athletes. Adolescence 36, 627–639. 11928872

[B61] Nicholls A. R. Madigan D. J. Backhouse S. H. Levy A. R. (2017b). Personality traits and performance enhancing drugs: The Dark Triad and doping attitudes among competitive athletes. Pers. Ind. Diffs. 112, 113–116. 10.1016/j.paid.2017.02.062

[B62] Nicholls A. R. Madigan D. J. Levy A. R. (2017a). A confirmatory factor analysis of the performance enhancement attitude scale for adult and adolescent athletes. Psych. Sport. Exerc. 28, 100–104. 10.1016/j.psychsport.2016.10.010

[B63] Nicholls A. R. Perry J. L. Levy A. R. Meir R. Jones L. Baghurst T. (2015). Coach perceptions of performance enhancement in adolescence: the Sport Drug Control Model for Adolescent Athletes. Perform. Enhance. Health 3, 93–101. 10.1016/j.peh.2015.07.001

[B64] Nicholls A. R. Taylor N. J. Carroll S. Perry J. L. (2016). The development of a new sport-specific classification of coping and a meta-analysis of the relationship between different coping strategies and moderators on sporting outcomes. Front. Psychol. 7:1674. 10.3389/fpsyg.2016.01674 27857697 PMC5093127

[B65] Nilsson S. (1995). Androgenic anabolic steroid use among male adolescents in Falkenberg. Eur. J. Clin. Pharmacol. 8, 9–11. 10.1007/bf00202164 7621856

[B66] Ntoumanis N. Ng J. Y. Y. Barkoukis V. Backhouse S. (2014). Personal and psychosocial predictors of doping use in physical activity settings: a meta-analysis. Sport. Med. 44, 1603–1624. 10.1007/s40279-014-0240-4 25138312

[B67] Origer A. Le Bihan E. Baumann M. (2014). Social and economic inequalities in fatal opioid and cocaine related overdoses in Luxembourg: A case-control study. Int. J. Drug Policy 25, 911–915. 10.1016/j.drugpo.2014.05.015 25002330

[B68] Paulhus D. L. Williams K. M. (2002). The Dark Triad of personality: Narcissism, Machiavellianism and psychopathy. J. Res. Pers. 36, 556–563. 10.1016/S0092-6566(02)00505-6

[B69] Pedersen W. Wichstrøm L. (2001). Adolescents, doping agents, and drug use: a community study. J. Drug. Issues. 31, 517–542. 10.1177/002204260103100208

[B70] Petróczi A. Aidman E. (2009). Measuring explicit attitude toward doping: review of the psychometric properties of the performance enhancement attitude scale. Psych. Sport. Exerc. 10, 390–396. 10.1016/j.psychsport.2008.11.001

[B71] Rees C. R. Zarco E. P. T. Lewis D. K. (2008). The steroids/sports supplements connection: pragmatism and sensation-seeking in the attitudes and behavior of JHS and HS students on Long Island. J. Drug. Educ. 38, 329–349. 10.2190/DE.38.4.b 19438066

[B72] Sagoe S. Mentzoni R. A. Hanss D. Pallesen S. (2016). Aggression is associated with increased anabolic–androgenic steroid use contemplation among adolescents. Subs. Use Misuse 51, 1462–1469. 10.1080/10826084.2016.1186696 27356242

[B73] Schirlin O. Rey G. Jouvent R. Dubal S. Komano O. Perez-Diaz F. (2009). Attentional bias for doping words and its relation with physical self-esteem in young adolescents. Psych. Sport. Exerc. 10, 615–620. 10.1016/j.psychsport.2009.03.010

[B74] Sedgwick P. (2014). Cross sectional studies. Advantages and disadvantages. BMJ 348:g2276 10.1136/bmj.g2276 25134102

[B75] Stilger V. G. Yesalis C. E. (1999). Anabolic-androgenic steroid use among high school football players. J. Comm. Health 24, 131–145. 10.1023/A:1018706424556 10202692

[B76] Terney R. McLain L. G. (1990). The use of anabolic steroids in high school students. Am. J. Dis. Child. 144, 99–103. 10.1001/archpedi.1990.02150250111046 2294728

[B77] Thorlindsson T. Halldorsson V. (2010). Sport, and use of anabolic androgenic steroids among Icelandic high school students: a critical test of three perspectives. Subst. Abuse. Treat. Prev. Policy 5:32. 21172024 10.1186/1747-597X-5-32 PMC3022538

[B78] vandenBerg P. Neumark-Sztainer D. Cafri G. Wall M. (2007). Steroid use among adolescents: longitudinal findings from project EAT. Pediatrics 119, 476–486. 10.1542/peds.2006-2529 17332200

[B79] Wanjek B. Rosendahl J. Strauss B. (2007). Doping, drugs and drug abuse among adolescents in the state of Thuringia (Germany): prevalence, knowledge and attitudes. Int. J. Sports. Med. 28, 346–353. 10.1055/s-2006-924353 17024651

[B80] Weiss M. R. Bredemeier B. J. (1983). Developmental sport psychology: a theoretical perspective for studying children in sport. J. Sport. Psych. 5, 216–230. 10.1123/jsp.5.2.216

[B81] Wichstrøm L. (2006). Predictors of future anabolic androgenic steroid use. Med. Sci. Sport. Exerc. 38, 1578–1583. 10.1249/01.mss.0000227541.66540.2f 16960518

[B82] Woolf J. Rimal R. N. Sripad P. (2014). Understanding the influence of proximal networks on high school athletes' intentions to use androgenic anabolic steroids. J. Sport. Man. 28, 8–20. 10.1123/jsm.2013-0046

[B83] World Anti-Doping Agency (WADA) (2015). World Anti-Doping Code. World Anti-Doping Agency QC, Canada.

[B84] Wroble R. R. Gray M. Rodrigo J. A. (2002). Anabolic steroids and preadolescent athletes: Prevalence, knowledge, and attitudes. Sport J. 5, 1–8.

[B85] Zelli A. Lucidi F. Mallia L. (2010a). The relationships among adolescents' drive for muscularity, drive for thinness, doping attitudes, and doping intentions. J. Clin. Sport. Psych. 4, 39–52. 10.1123/jcsp.4.1.39

[B86] Zelli A. Mallia L. Lucidi F. (2010b). The contribution of interpersonal appraisals to a social-cognitive analysis of adolescents' doping use. Psych. Sport Exerc. 11, 304–311. 10.1016/j.psychsport.2010.02.008

